# A Whole Recombinant Yeast-Based Therapeutic Vaccine Elicits HBV X, S and Core Specific T Cells in Mice and Activates Human T Cells Recognizing Epitopes Linked to Viral Clearance

**DOI:** 10.1371/journal.pone.0101904

**Published:** 2014-07-22

**Authors:** Thomas H. King, Charles B. Kemmler, Zhimin Guo, Derrick Mann, Yingnian Lu, Claire Coeshott, Adam J. Gehring, Antonio Bertoletti, Zi Z. Ho, William Delaney, Anuj Gaggar, G. Mani Subramanian, John G. McHutchison, Shikha Shrivastava, Yu-Jin L. Lee, Shyamasundaran Kottilil, Donald Bellgrau, Timothy Rodell, David Apelian

**Affiliations:** 1 GlobeImmune, Inc., Louisville, Colorado, United States of America; 2 Integrated Department of Immunology, University of Colorado School of Medicine, Aurora, Colorado, United States of America; 3 Gilead Sciences Inc., Foster City, California, United States of America; 4 Molecular Microbiology and Immunology & Saint Louis University Liver Center, Saint Louis University School of Medicine, Saint Louis, Missouri, United States of America; 5 Agency for Science, Technology and Research (A*STAR), Singapore Institute for Clinical Sciences, Singapore, Singapore; 6 Laboratory of Immunoregulation, National Institute of Allergy and Infectious Diseases, National Institutes of Health, Bethesda, Maryland, United States of America; National Institute of Allergy and Infectious Diseases, United States of America

## Abstract

Chronic hepatitis B infection (CHB) is characterized by sub-optimal T cell responses to viral antigens. A therapeutic vaccine capable of restoring these immune responses could potentially improve HBsAg seroconversion rates in the setting of direct acting antiviral therapies. A yeast-based immunotherapy (Tarmogen) platform was used to make a vaccine candidate expressing hepatitis B virus (HBV) X, surface (S), and Core antigens (X-S-Core). Murine and human immunogenicity models were used to evaluate the type and magnitude of HBV-Ag specific T cell responses elicited by the vaccine. C57BL/6J, BALB/c, and HLA-A*0201 transgenic mice immunized with yeast expressing X-S-Core showed T cell responses to X, S and Core when evaluated by lymphocyte proliferation assay, ELISpot, intracellular cytokine staining (ICS), or tumor challenge assays. Both CD4^+^ and CD8^+^ T cell responses were observed. Human T cells transduced with HBc18–27 and HBs183–91 specific T cell receptors (TCRs) produced interferon gamma (IFNγ following incubation with X-S-Core-pulsed dendritic cells (DCs). Furthermore, stimulation of peripheral blood mononuclear cells (PBMCs) isolated from CHB patients or from HBV vaccine recipients with autologous DCs pulsed with X-S-Core or a related product (S-Core) resulted in pronounced expansions of HBV Ag-specific T cells possessing a cytolytic phenotype. These data indicate that X-S-Core-expressing yeast elicit functional adaptive immune responses and supports the ongoing evaluation of this therapeutic vaccine in patients with CHB to enhance the induction of HBV-specific T cell responses.

## Introduction

Chronic hepatitis B virus infection (CHB) is a major worldwide public health concern. An estimated two billion people worldwide show serological evidence of past or present hepatitis B virus (HBV) infection and an estimated 400 million people are chronically infected [1]. About 25% of CHB patients ultimately develop hepatic decompensation, liver cirrhosis or hepatocellular carcinoma and more than one million people die annually from these complications [2].

Most approved approaches to treating CHB are aimed at prevention (e.g., immunization with prophylactic vaccines that generate humoral responses), or controlling viral replication with drugs such as tenofovir disoproxil fumarate (TDF), entecavir, lamivudine, or interferon-alpha (IFN-α) (reviewed in [3]). The nucleos(t)ide analog-based polymerase inhibitors such as entecavir and TDF effectively inhibit HBV genome replication, but result in the loss of HBsAg (HBsAg seroconversion) in less than 10% of subjects after many years of therapy, requiring life-long treatment to maintain viral suppression [4], [5], [43]. These considerations underscore the need for improved therapies for CHB that are safe and able to provide durable immune control and enhance rates of HBsAg seroconversion with a finite treatment duration.

The low rate of HBsAg seroconversion that is achieved with the current treatments is partially attributed to insufficient HBV-specific T cell responses [6]. HBV-infected cells possess a stable pool of covalently closed circular viral DNA (cccDNA) that is a reservoir for viral replication and antigen production. Thus, suppression of viral replication based on inhibition of viral enzymes without concomitant T cell-mediated elimination of infected hepatocytes is insufficient to effect durable off-treatment control of the disease. In acute self-limited disease, the adaptive immune system elicits polyclonal and multi-antigen specific T-cell responses as well as type 1 interferon that are critical in the antiviral response and result in both non-cytolytic silencing and cytolytic elimination of cells containing HBV [7]–[9], [44]. In CHB patients, the breadth and magnitude of these immune responses are reduced and the antigen specificity is narrow. These effects are due to interrelated factors including central and/or peripheral tolerance mechanisms, ineffective immune priming, T cell exhaustion, and regulatory T cell imbalance [10]–[13]. An immunotherapeutic approach capable of overcoming any or all of these immune deficiencies could potentially improve viral clearance rates in CHB.

We have developed an immunotherapeutic platform called Tarmogen (Targeted Molecular Immunogen) therapy that is comprised of heat-inactivated, whole recombinant *Saccharomyces cerevisiae* yeast cells expressing disease-related antigens. This vector can deliver multiple antigens into the MHC class I and II antigen presentation pathways to stimulate potent CD4^+^ and CD8^+^ T cell responses [14], and can break immunological tolerance to tumor antigens in transgenic mouse models [15]. The yeast vector is also not readily neutralized *in vivo* and is therefore amenable to repeated administration, enabling the application of long-term immunological pressure, ideal for the elimination of chronic intracellular infections such as HCV and HBV [16]. Recent work has also shown that the Tarmogen platform triggers a reduction in the number and immunosuppressive activity of regulatory T cells [17], likely due to the natural ability of yeast to elicit IL-1β production and Th17 T cell differentiation at the expense of regulatory T cells [18].

An analogous Tarmogen product expressing a HCV NS3-Core fusion protein (GI-5005) was evaluated in an open label phase 2 clinical trial comparing standard of care treatment (SOC: type I IFN and ribavirin) to SOC combined with GI-5005 in 140 patients chronically-infected with genotype 1 HCV. The results showed a statistically significant 15% overall improvement in end-of-treatment response and notably, in a cohort of patients that harbor the IL28B rs12979860 allele T/T, a genotype that is notably refractory to SOC treatment [19], a benefit in complete virologic response was observed for the GI-5005 group compared to SOC alone (63% for GI-5005+SOC vs. 27% for SOC alone [20]. GI-5005 showed comparable rates of serious adverse events compared to SOC and the Tarmogen platform as a whole has been well-tolerated, with safety data in >400 patients accrued over 12 human clinical trials.

Based on the Tarmogen platform's characteristics and the clinical activity observed in chronic HCV patients, we hypothesized that a Tarmogen designed to express HBV antigens could be an ideal candidate for CHB immunotherapy. A panel of Tarmogens expressing varied combinations of the 4 major HBV antigens was created and tested for antigen expression and growth. Among these, one emerged as a promising lead for immunogenicity testing: a fusion protein expressing the most highly conserved regions of HBxAg, HBsAg, and HBcAg (X-S-Core or GI-13020/GS-4774). This study evaluated the immunogenicity of GS-4774 in murine and human *ex vivo* T cell models, as a proof-of-concept justifying assessment in human clinical trials for the treatment of CHB.

## Results

### Selection of a lead candidate HBV therapeutic vaccine

A panel of Tarmogens was created featuring fusion proteins comprised of conserved protein domains of HBV X, S, Core and Polymerase reverse transcriptase domain (Pol) antigens. Expression and growth analyses identified two candidates: a S-Core fusion (S-Core) and an X-S-Core fusion (X-S-Core) that possessed high level antigen expression and growth properties compatible with larger scale production. Candidates containing Pol consistently expressed low levels of antigen and were excluded from further analyses. Multiple preliminary immunogenicity assays (not shown) determined that X-S-Core (GS-4774) was more immunogenic than S-Core in mice, and GS-4774 was thus chosen as the lead candidate for full experimental evaluation.

**Figure 1:**
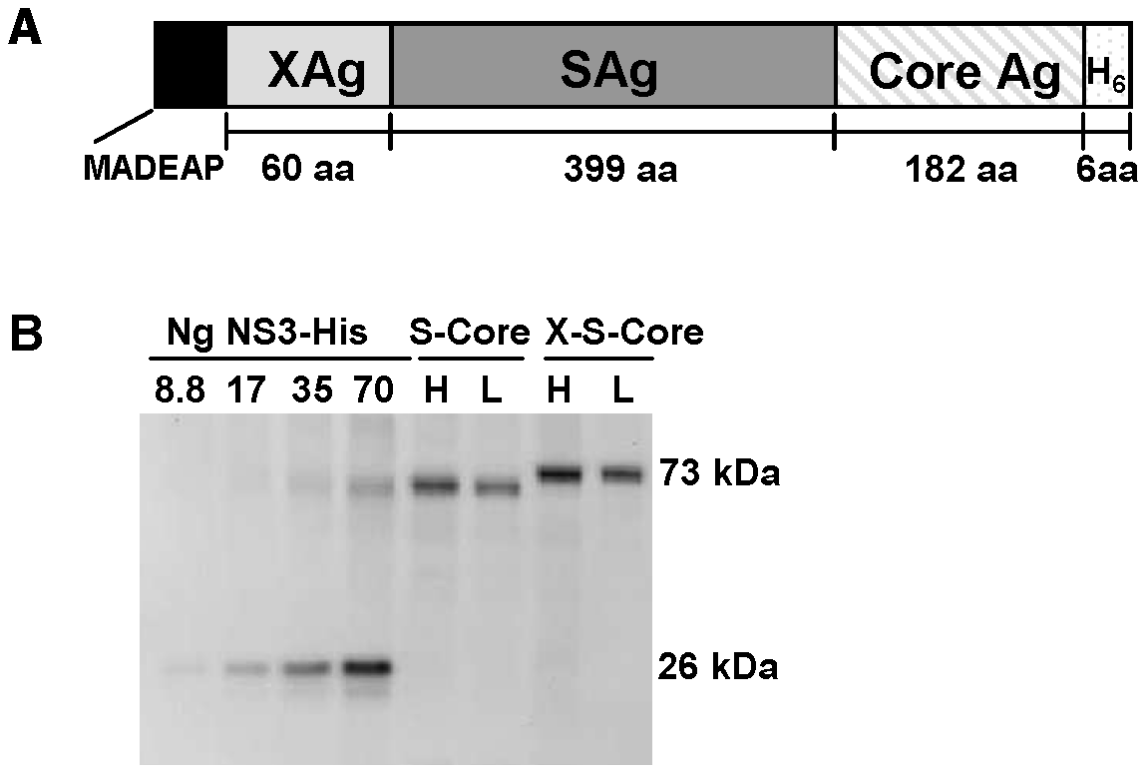
Structure and expression of the X-S-Core fusion protein expressed in the GS-4774 Tarmogen. (A) Schematic representation of the ∼73 kDa HBV X-S-Core fusion protein. MADEAP: sequence imparting metabolic stability in yeast; XAg: 60 (non-contiguous) amino acids of the HBxAg; SAg: 399 contiguous amino acids of HBsAg (entirety of large SAg except for N terminal methionine); Core Ag: 182 contiguous amino acids of HBcAg, lacking the N-terminal methionine; H_6_ hexahistidine epitope tag. (B) Western blot probed with anti-his tag mAb, showing expression of the S-Core and X-S-Core fusion proteins in GS-4774 yeast. NS3-his std: purified recombinant, his tagged HCV NS3 protein for quantification of X-S-Core, H and L: high (6 µg) and low (3 µg) levels of total protein loaded per lane.

The structure of the HBV Ag expressed in GS-4774 features conserved domains of HBV genotype D consensus sequences for the X, S and Core antigens (Fig 1A). The expressed regions of these antigens are 88 to 99% identical to the corresponding domains of HBV genotypes A–C, consistent with the potential of this vaccine to elicit activity across the four major HBV genotypes. The X-S-Core fusion accumulates to approximately 2000 ng antigen per YU (YU:10^7^ yeast cells) and migrates at approximately 73 kDa (Fig 1B). The S-Core fusion accumulates to approximately 1450 ng per YU and migrates at 66 kDa. Two different levels of loaded total protein per lane are shown for each Tarmogen (see Figure legend for details).

### GS-4774 immunization elicits lymphocyte proliferation in mice

To evaluate whether GS-4774 immunization expands antigen-specific T cells in mice, a lymphocyte proliferation assay (LPA) was conducted. This assay can be used to assess the antigen specificity of the response and to determine the T cell subset(s) involved. The former is assessed by varying the antigens added to in vitro stimulation and by vaccinating with control Tarmogens; the latter by using cell preparations that are highly enriched for a T cell subset(s) (CD4^+^ in this study).

**Figure 2:**
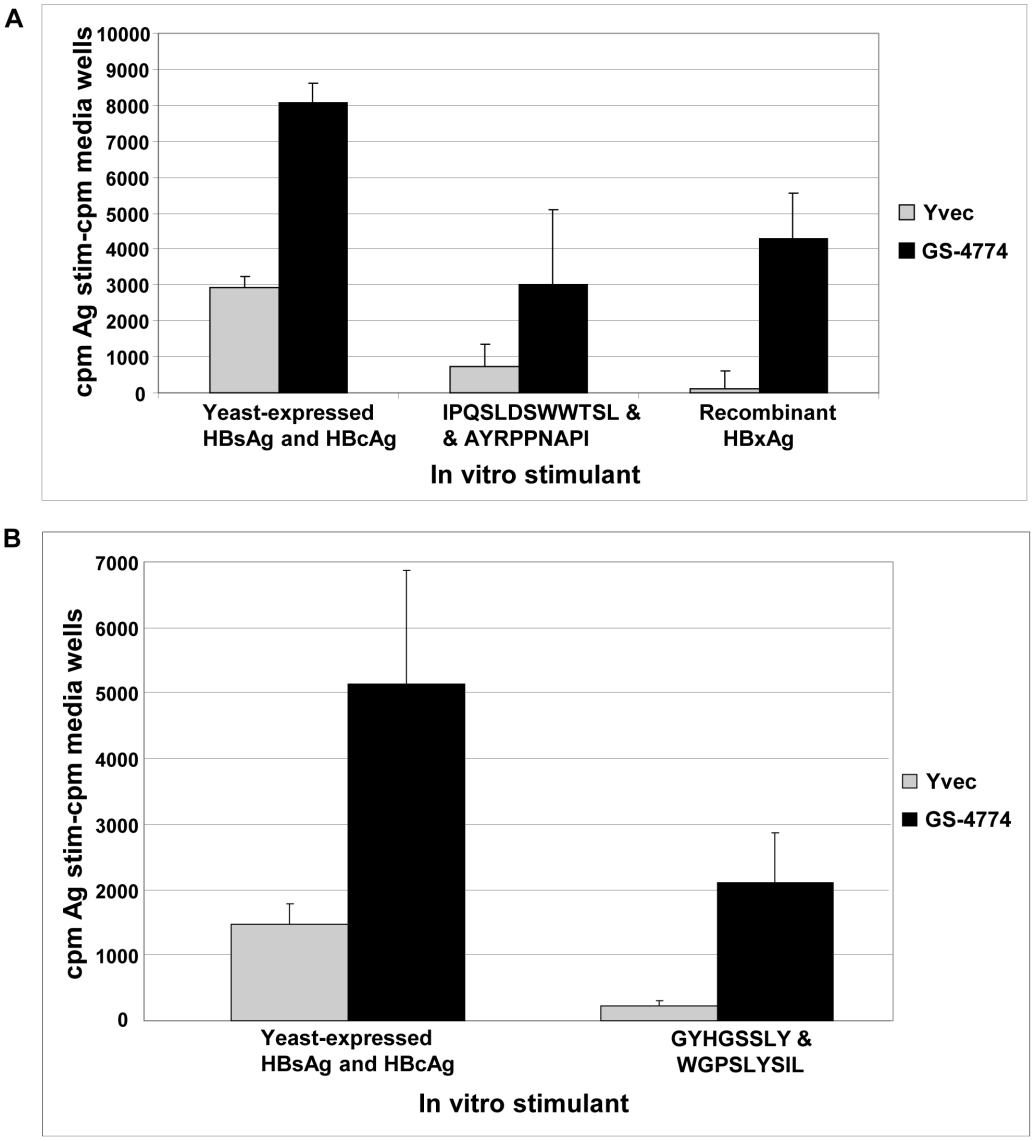
Ex vivo lymphocyte proliferation assays of T cells from GS-4774-immunized mice. (A) Proliferation of inguinal LN cells harvested from GS-4774- or Yvec-immunized BALB/c mice. LN cells from 5 immunized mice were pooled and placed into in vitro stimulation with the indicated antigens for 4 days, followed by a 20 h ^3^H-thymidine uptake assay. In vitro stimulants are: *Pichia pastoris* yeast expressed, purified HBsAg and HBcAg, 3 µg/mL each; IPQSLDSWWTSL (HBsAg L^d^ restricted peptide) and AYRPPNAPI (HBcAg L^d^ restricted peptide), 10 µg/mL each; recombinant *E. coli-* expressed full length HBxAg, 3 µg/mL. **P values**, GS-4774 vs. Yvec: yeast expressed HBsAg & HBcAg, 0.0001; IPQSLDSWWTSL & AYRPPNAPI peptides, 0.089; recombinant HBxAg, 0.002. (B) Proliferation of CD4^+^ T cells isolated from splenocytes of GS-4774- or Yvec-immunized BALB/c mice. MACS-isolated splenic CD4^+^ T cells were stimulated with the indicated HBV antigens and assayed as in (A). Yeast expressed HBsAg & HBcAg stimulants: same as for panel A; GYHGSSLY, a MHC class II HBsAg mimetic peptide plus WGPSLYSIL, a 2-D^d^ restricted HBsAg peptide (10 µg/mL each). **P values**, GS-4774 vs. Yvec: yeast expressed HBsAg & HBcAg, 0.034; GYHGSSLY and WGPSLYSIL, 0.023. ^3^H-thymidine uptake is reported with medium background subtracted counts per minute (cpm) for each antigen stimulation condition. Error bars: standard error (s.e.) for replicate stimulations of the pooled immune cells.

BALB/c mice were subcutaneously (s.c.) immunized in the flank and in the scruff of the neck with 2.5 YU of Tarmogen per site (method A, see “Mice and Immunization” for details) with GS-4774 or empty vector yeast (Yvec) control and their spleens or lymph nodes (LNs) were removed and stimulated *in vitro* with HBV peptides and recombinant antigens. Lymphocyte proliferation assays of cells stimulated *in vitro* showed that GS-4774 elicited T cell proliferative responses in mice that were specific for each individual HBV antigen: X, S and Core (Fig 2A). This assay was also used to demonstrate a significant CD4^+^ T cell contribution in this antigen-specific response, as a 3.5 to 9.2-fold Tarmogen/Yvec response ratio was observed using a splenic CD4^+^ T cell preparation of >90% purity (Fig. 2B and not shown). We note that one of the peptides used in the pool (Fig. 2B right, WGPSLYSIL) was previously published to be a MHC-cI-restricted epitope and as such may not have contributed to the recall response. Other cell types such as CD8^+^ T cells could have contributed to the proliferative signal in Fig. 2B, although probably in a minor way given the relatively high purity of CD4^+^ T cell preparation. We note that the weight and overall health of the mice used in this and all subsequent murine experiments was 15–19 g, and mice were healthy and specific pathogen free at baseline. No adverse events were found during Tarmogen treatment regimens. All mice in each cohort were evaluated in establishing conclusions and statistical summaries.

### GS-4774 induces T cells that secrete Th1-type, pro-inflammatory cytokines IFNγ and IL-2

**Figure 3:**
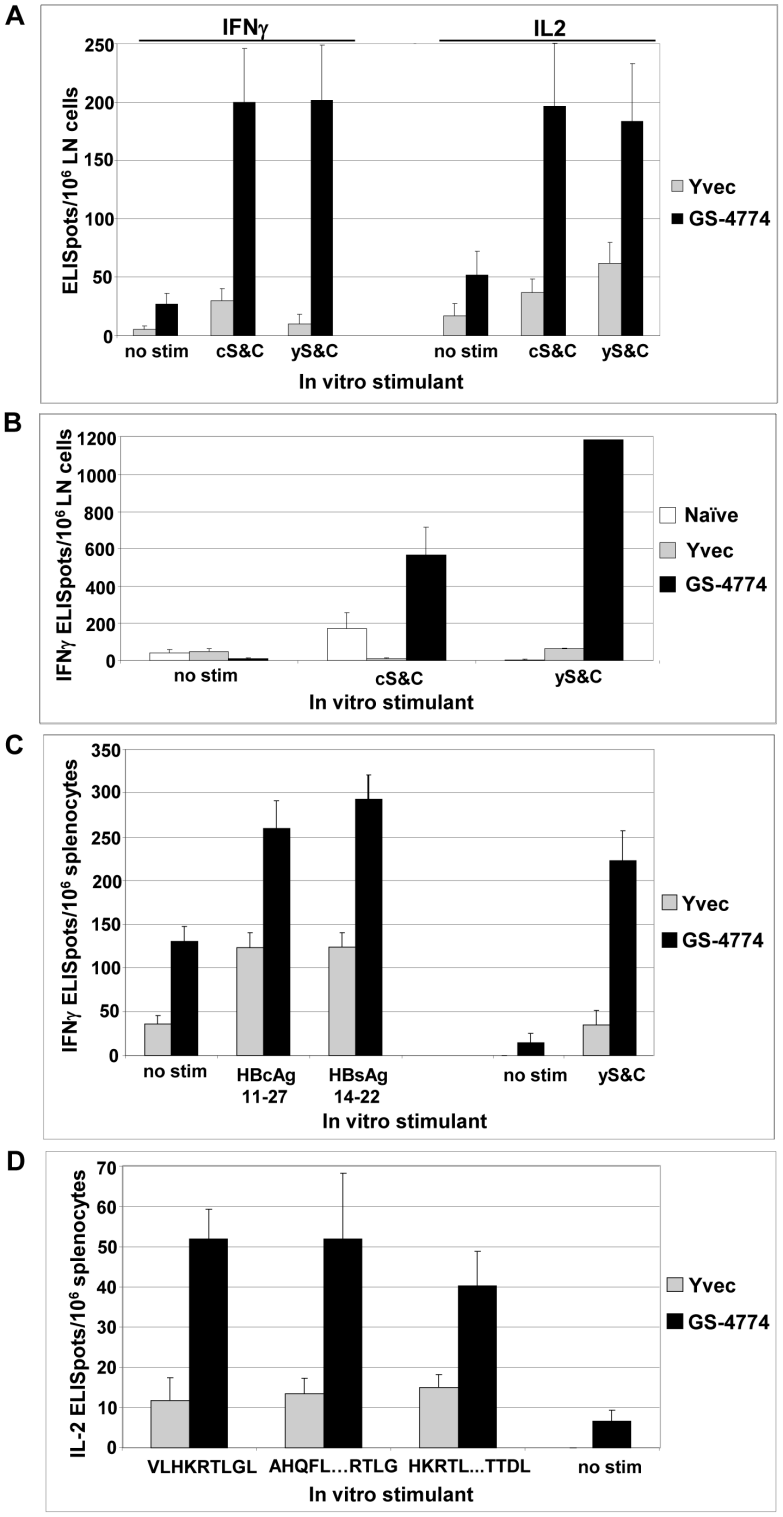
IFNγ and IL2 ELISpot responses in GS-4774-immunized mice. (A) LN cells from 5 pooled GS-4774- or 5 pooled Yvec-immunized BALB/c mice were stimulated with a 1∶1 mixture of *E. coli* or *Pichia pastoris* yeast-expressed HBsAg and HBcAg (cS&C, and yS&C, 3 µg/mL each). Control “no stim” wells contained growth medium plus 10% FBS only. Left, IFNγ; right, IL2. **P values**, GS-4774 vs. Yvec: cS&C-IFNγ, 0.0001; yS&C-IFNγ, 0.001; cS&C-IL2, 0.004; yS&C-IL2, 0.027. (B) IFNγ ELISpot response in GS-4774-or Yvec-immunized C57BL/6 mice. LN cells of 5 immunized mice per group were pooled and stimulated as in (A). **P values**, GS-4774 vs. Yvec: cS&C, 0.020; yS&C, 0.0081. (C) IFNγ ELISpot responses specific for acute-resolved, MHC class I restricted epitopes in GS-4774- or Yvec-immunized HLA-A*02∶01 tg mice (n = 5 mice per group). HBcAg 11–27: ATVELLSFLPSDFFPSV; HBsAg 14–22: VLQAGFFLL; yS&C and no stim, see panel A. (D) IL2 ELISpot responses to novel HBxAg epitopes in BALB/c mice. Splenocytes from 10 immunized mice were pooled and stimulated with 7 µM of 44 different 9-mer and 32 different 15-mer peptides for 4 days followed by a 24 h IL2 ELISpot assay. Only positive responding peptides are shown here (see also Fig. S2 for full results) and are defined as those with > 40 spots per million splenocytes in GS-4774 immunized mice and for which the GS-4774/Yvec response ratio was >2.5. Sequences of positive responding peptides: VLHKRTLGL, AHQFLPKVLHKRTLG or HKRTLGLSAMSTTDL. **P values**, GS-4774 vs. Yvec: VLHKRTLGL, 0.005; AHQFLPKVLHKRTLG, 0.061; HKRTLGLSAMSTTDL, 0.034. Error bars: s.e. for replicate stimulations of the pooled immune cells.

IFNγ and IL-2 are produced by activated T cells in the effector phase of an adaptive immune response, and are subsequently used to assess the efficiency of Th1 T cell induction. To evaluate whether GS-4774 elicits HBV Ag-specific T cells capable of producing these cytokines, BALB/c mice were immunized by method A with GS-4774 or Yvec and LN cells were subjected to IFNγ/IL-2 dual color ELISpot assays. The results showed that GS-4774 immunization elicited T cells capable of producing IFNγ and/or IL-2 upon re-stimulation with *E.coli* or *Pichia*-expressed recombinant HBV antigens. GS-4774 immunization produced significantly stronger signals than did immunization with the Yvec control, demonstrating the HBV Ag-specificity of the response (Figure 3A; Tarmogen/Yvec response ratio of up to 20.2). The experiment was also conducted with C57BL/6 mice in which in vitro stimulation with recombinant S and Core antigens elicited a rigorous IFNγ response in GS-4774-immunized mice that was up to 57-fold greater than that of Yvec-control immunized mice (Fig. 3B).

As these responses in prior experiments were in the context of murine MHC, we next evaluated the immunogenicity of GS-4774 in the context of HLA-A*02∶01 (HLA-A2), a common human allele in the HBV-infected population. HLA-A2 transgenic (tg) mice were immunized with GS-4774 or Yvec per method A. Two weeks after the last immunization, splenocytes were harvested and stimulated *in vitro* with HBV peptides corresponding to epitopes of known importance in the clearance of acute HBV infection: HBcAg 11–27 and HBsAg 14–22 [21]. Stimulations were also done with purified recombinant HBsAg and HBcAg as an additional means of assessing the response of vaccine-emergent T cells to HBV antigens more generally, presented in the context of HLA-A2 and/or endogenous murine MHC. After a 4 day in vitro stimulation, cells were evaluated for IFNγ production by ELISpot. Cells stimulated with the HBV peptides or the recombinant antigens produced IFNγ with spot frequencies up to 280 per million splenocytes. Tarmogen/Yvec response ratios of up to 6.4 were observed (Fig. 3C).

These ELISpot results address the immunogenicity of S and Core antigens but not the HBxAg component of GS-4774. Literature searches did not identify HBxAg-specific peptides suitable for use in murine experiments, so we undertook an approach to empirically identify novel HBxAg epitopes in GS-4774-immunized mice. A panel of 44 9-mer and 32 15-mer peptides was created from the HBxAg sequence, to represent selected domains of the 60 amino acids of HBxAg that are present in GS-4774. The 9-mer and 15-mer lengths were chosen to favor the selection of MHC class I and class II epitopes, respectively. BALB/c mice were immunized by method A, with Yvec or GS-4774, rested for 11 days, and then boosted once more. One week post-boost, splenocytes were placed into in vitro stimulation with each peptide for 4 days and then cells were subjected to IFNγ/IL-2 dual color ELISpot analysis. The results showed that IL-2 was produced in response to one of the 9-mer peptides (VLHKRTLGL) and two of the 15-mer peptides (AHQFLPKVLHKRTLG and HKRTLGLSAMSTTDL). Positive responses in this experiment were defined as those with >40 spots per million splenocytes in GS-4774-immunized mice and for which the GS-4774/Yvec response ratio was at least 2.5. P values for the GS-4774/Yvec comparison for these 3 peptides (ANOVA) ranged from 0.06 to 0.005 (see also Fig. 3D legend). HBV-Ag specificity was indicated by the Tarmogen/Yvec response ratios of 2.7 through 4.4 for these peptides (Fig. 3D). For the sequences of all HBX peptides screened, see Figure S1 and for ELISpot responses for all HBxAg peptides tested see Figure S2.

Consistent with the LPA results, these ELISpot data demonstrate that GS-4774 immunization induces Th1-type immune responses to all 3 HBV antigens contained in the X-S-Core fusion. The S and Core epitope-specific T cells observed in HLA-A2 tg mice are consistent with the potential of GS-4774 to elicit HBV clearance in humans, based on the presence of these T cell specificities in individuals who clear acute infection without therapeutic intervention.

### GS-4774 immunization induces HBsAg and HBcAg-specific CD4^+^ and CD8^+^ T cell responses

As CD8^+^ T cells are the effector T cell population most likely to result in clearance of virally infected cells, and because CD4^+^ T cells provide important help to achieve optimal CD8^+^ T cell responses, we used ICS to determine if HBV Ag-specific CD4^+^ and CD8^+^ T cells are generated by GS-4774 immunization. Pooled splenocytes from 7 GS-4774-vaccinated C57BL/6 mice were incubated with peptide HBcAg 120-140 VSFGVWIRTPPAYRPPNAPIL or HBsAg 190–197 VWLSVIWM, each previously shown by others to be active in murine immunogenicity assays [22], [23]. The peptide-expanded T cells were subjected to intracellular cytokine staining (ICS) to evaluate cytokine production by CD4^+^ (IFNγ) and CD8^+^ T cells (IFNγ plus IL-2 or IFNγ plus TNFα). The results indicated that GS-4774 but not Ovax (control yeast) immunization elicited HBs190–197 specific CD8^+^ T cells producing IFNγ plus IL-2, or IFNγ plus TNFα following HBs190–197 stimulation (Table 1 top panel; for sample flow cytometry data see Fig. S3). We note that Ovax was used this and in several subsequent experiments because it was reasoned that a Tarmogen expressing an irrelevant antigen (chicken ovalbumin) could be considered more meaningful than one expressing no heterologous antigen (Yvec), although in practice the two Tarmogens produce a similar and level of background immune stimulation. GS-4774/Ovax response ratios for the CD8^+^ T cell subset ranged from 3.3 to 6.4, demonstrating a requirement for the X-S-Core Ag for a HBV-specific CD8^+^ T cell response. T cells producing these multiple cytokines simultaneously are believed to be better equipped to control viral infection than those producing IFNγ alone [9], [24], [25]. CD4^+^ T cell responses were also observed although the number of Ag specific cells and the response ratio (1.9) were lower than for the CD8^+^ T cell population (Table 1 bottom panel).

**Table 1:** Th1 cytokine responses in CD4^+^ and CD8^+^ T cells from GS-4774 vs. Ovax-immunized C57BL/6 mice.

Vaccine	% of total CD8^+^ T cells following VWLSVIWM stimulation		
	IFNγ^+^	IFNγ^+^IL2^+^	IFNγ^+^TNFα^+^
GS-4774	8.2 (0.0001)	1.4 (0.0001)	1.9 (0.0001)
Ovax	2.5	0.22	0.53
GS-4774/Ovax ratio	3.3	6.4	3.6

Vaccine	% IFNγ^+^ of CD4^+^ T cells after VSFGVWIRTPPAYRPPNAPIL stim.	
	0.5 µg/mL peptide	5 µg/mL peptide
GS-4774	0.40	0.51
Ovax	0.14	0.14
GS-4774/Ovax ratio	2.9	3.6

Intracellular cytokine staining was used to assess the production of IFNγ, IL2, and TNFα by CD8^+^ T cells in the presence of peptide HBs190–197 (VWLSVIWM; top), and the production of IFNα in CD4^+^ T cells in the presence of peptide HBcAg 120–140 VSFGVWIRTPPAYRPPNAPIL (bottom). **P values**: in parentheses. n.t., not tested. Ovax: control Tarmogen expressing chicken ovalbumin. Splenocytes from 7 vaccinated mice per group were pooled for the analysis.

### GS-4774 protects mice from challenge with syngeneic EL4 tumors expressing HBV antigens

To determine if the immune response elicited by GS-4774 can clear HBV Ag-expressing cells *in vivo*, tumor challenge studies were conducted. Two general models were used: i) adoptive transfer in which T cells from immunized donor mice were transferred to recipient severe combined immuno- deficiency (*scid*) mice prior to tumor challenge, and; ii) a classical prophylactic approach in which wt mice were immunized and then challenged *in situ*. These models were conducted with immunization at each of 4 sites, with 1 YU of Tarmogen per site (method B, see Methods section “Mice and Immunization” for details of the regimen). This immunization method was shown to be superior to method A for a murine tumor challenge application with another Tarmogen [15].

The adoptive transfer method showed that immunization with GS-4774 elicited HBV Ag-specific lymphocytes that adoptively inhibited growth of EL4 tumors expressing a fusion of HBsAg and HBcAg (Table 2, top row; p = 0.00029).

**Table 2:** GS-4774 immunization inhibits growth of syngeneic, HBV-Ag expressing tumors in mice.

Tumor	Vaccine	Diameter (mm)	Ovax/GS-4774 ratio	Mice with tumors
EL4/S-Core-Adoptive (day 7)	Ovax	7.4	4.1	8/8
	GS-4774	1.8		3/8 (p = 0.00029*)
EL4/S-Core (day 8)	Ovax	4.9	4.1	6/9
	GS-4774	1.2		2/10 (p = 0.004)
EL4/X (day 9)	Ovax	7.1	3.1	10/10
	GS-4774	2.3		8/10 (p = 0.006)
EL4/Core (day 10)	Ovax	5.6	2.1	12/14
	GS-4774	2.7		7/14 (p = 0.009)

C57BL/6 mice (n = 8 per group) were thrice immunized and then challenged s.c. with syngeneic EL4 tumors expressing HBV antigens. Tumor diameters correspond to size at days 7, 8, 9, or 10 post-challenge. EL4/S-Core-Adoptive (day 7): data are from adoptive transfer of splenocytes from GS-4774- or Ovax-immunized mice to *scid* recipients prior to tumor challenge. “Mice with tumors”: fraction of mice with measureable tumors at the indicated day.

*P-values: applicable to comparison of tumor diameters for GS-4774 vs. Ovax for each experiment (ANOVA).

**Figure 4:**
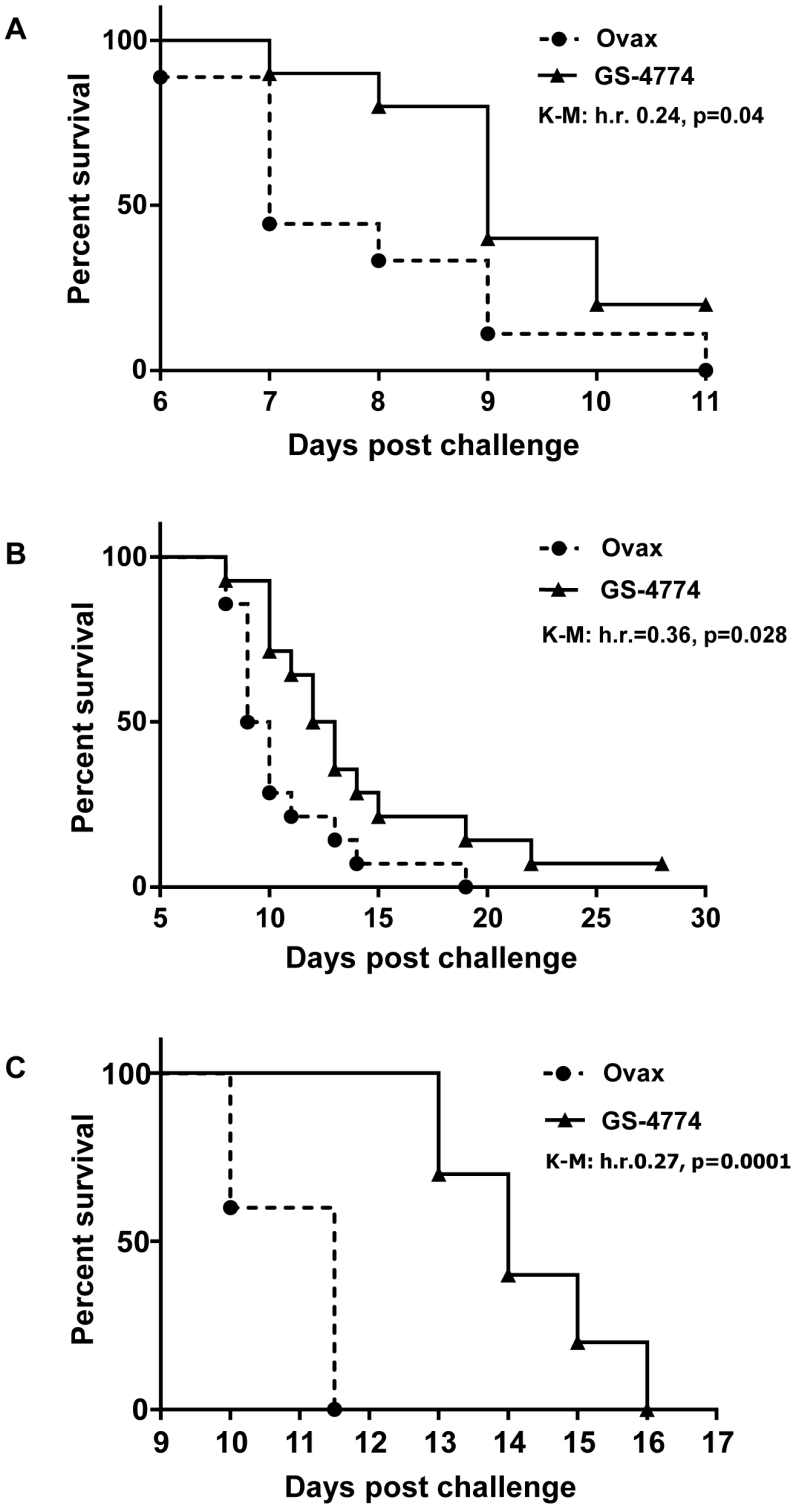
GS-4774 immunization inhibits growth of syngeneic, HBV-Ag expressing tumors in mice. C57BL/6 mice were thrice immunized and then challenged s.c. with syngeneic EL4 tumors expressing HBV antigens. Kaplan-Meier analyses correspond to mice challenged with tumors expressing: (A) S-Core fusion (n = 10 recipient mice per group); (B) HBcAg (n = 14/group), or; (C) HBxAg (n = 10/group). hr, hazard ratio (hazard rate GS-4774/hazard rate Ovax; see methods). **P values** : see Figure. Ovax: Control Tarmogen expressing chicken ovalbumin.

The prophylactic approach was then used in three experiments, featuring EL4 tumors expressing the S-Core fusion, HBcAg, or HBxAg at challenge doses of 300,000, 30,000, and 90,000 EL4 cells, respectively. GS-4774 conferred statistically significant protection against these tumors, although to different extents depending on the target evaluated (see Figures 4A-C for Kaplan-Meier survival format). For example, growth of the HBxAg- and S-Core fusion expressing tumor cells was somewhat more strongly inhibited by the immunization (hazard ratios [HR] of 0.27, and 0.24; p values 0.0001 and 0.04, respectively) than the HBcAg-expressing target (HR 0.36, p = 0.028). The tumor size data also show significant protective effects of GS-4774, with Ovax/GS-4774 tumor diameter ratios ranging from 4.1 to 2.1 depending on the target cell evaluated (Table 2). We were unable to conclusively evaluate HBsAg-specific protection because attempts to engineer EL4 target cells expressing HBsAg were unsuccessful. It is noted that C57BL/6 mice immunized with Ovax were protected from challenge with EL4 cells expressing ovalbumin (E.G7/Ova) in a previously published study [14]. In other experiments, a closely related Tarmogen (S-Core) protected mice from challenge with EL4 tumors expressing S-Core Ag but not E.G7/Ova target cells (Figure S4). The tumor protection conferred by the HBV Tarmogens was not 100% as shown in the K-M graphs. This prompted us to ask whether tumors that escaped immune-mediated killing had retained HBV antigen expression, because EL4 cells lacking HBV antigens are not expected to be targets of GS-4774-induced T cells. Tumors that escaped immune-mediated killing were excised from mice and the level of S-Core mRNA was measured by real time PCR. The results showed that as few as ∼3% of EL4 cells within these tumors had detectable S-Core mRNA using this sensitive assay, i.e., up to 97% of tumor cells that escaped the immune response were devoid of detectable HBV antigen (Figure S5). Together with the results shown in Figure S4, these data collectively indicate that protection from HBV-Ag expressing tumors requires HBV Ag expression in both the vaccine and in the tumor target, and that the lack of complete tumor protection is likely explained in large part by outgrowth of EL4 cells lacking HBV antigen expression. The findings provide evidence of the capacity of GS-4774 to elicit HBV-targeted responses *in vivo*.

### GS-4774-treatment elicits efficient maturation of human DCs

To extend the analysis to human cells, *ex vivo* tests were performed in which DCs prepared from healthy or HBV Ag-vaccinated donors were treated with GS-4774 and then used to stimulate autologous T cells.

We first determined if GS-4774 elicits maturation of human DCs. Immature monocyte-derived DCs (imoDCs) were incubated with GS-4774 and then analyzed by flow cytometry using dye-coupled antibodies recognizing established DC maturation markers.

**Figure 5:**
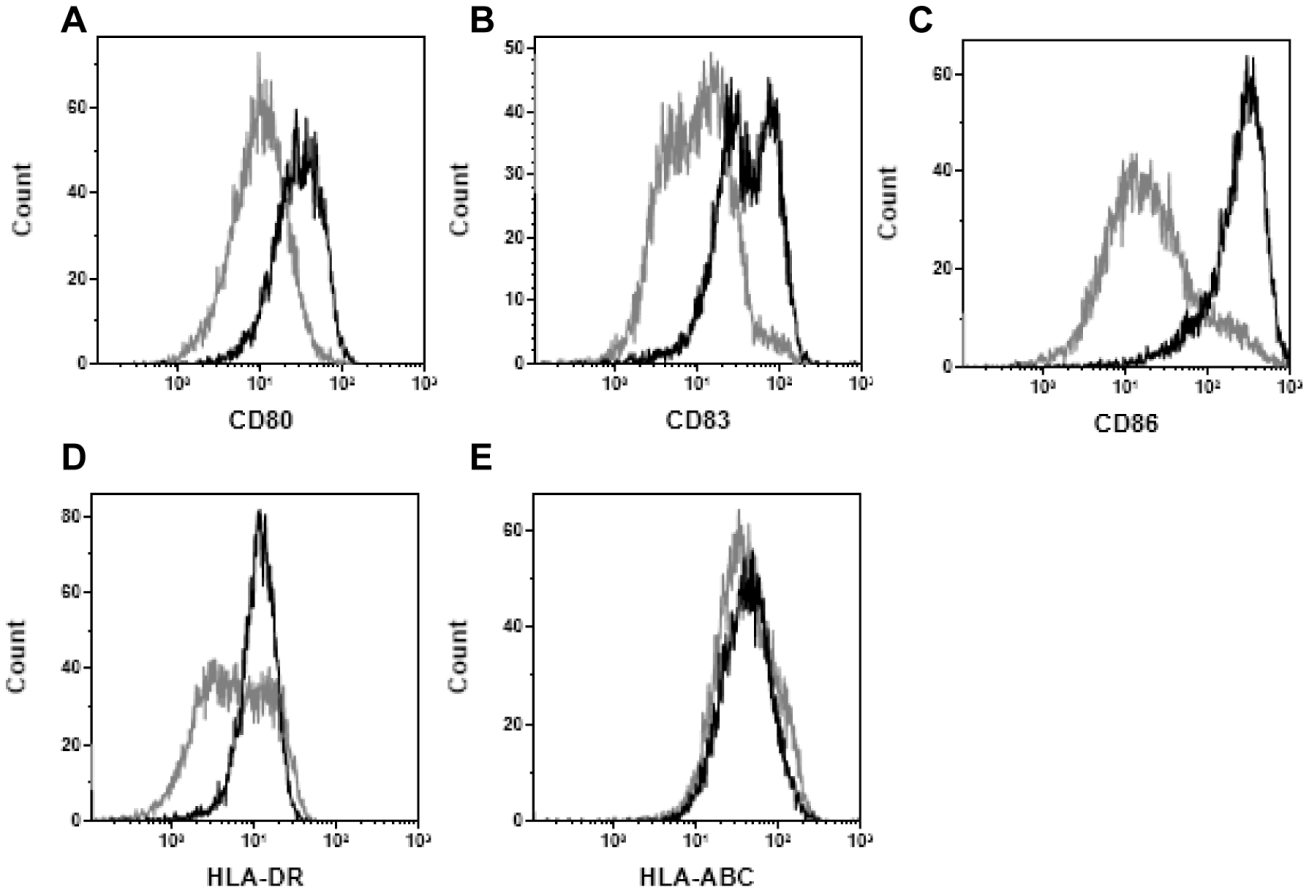
GS-4774 induces maturation of human monocyte-derived dendritic cells (moDCs). CD14^+^ monocytes were isolated from healthy donors and cultured with GM-CSF + IL-4 for 6 days to generate immature moDCs which were then incubated for 24 h with 10 Tarmogens per 1 moDC. The moDCs were stained with dye-coupled antibodies recognizing A, CD80; B, CD83; C, CD86; D, HLA-DR or; E, HLA-A, B, & C and evaluated by flow cytometry.

GS-4774 elicited an increase in all of the maturation markers with mean fluorescence intensity increases of up to ∼1.2 log_10_ (Fig. 5). Similar results were shown for a closely related Tarmogen expressing HBV S-Core fusion (Fig. S6). These data confirmed that GS-4774 triggered DC maturation as reported for other Tarmogens in human and mouse cells [14], [26], [27].

**Figure 6:**
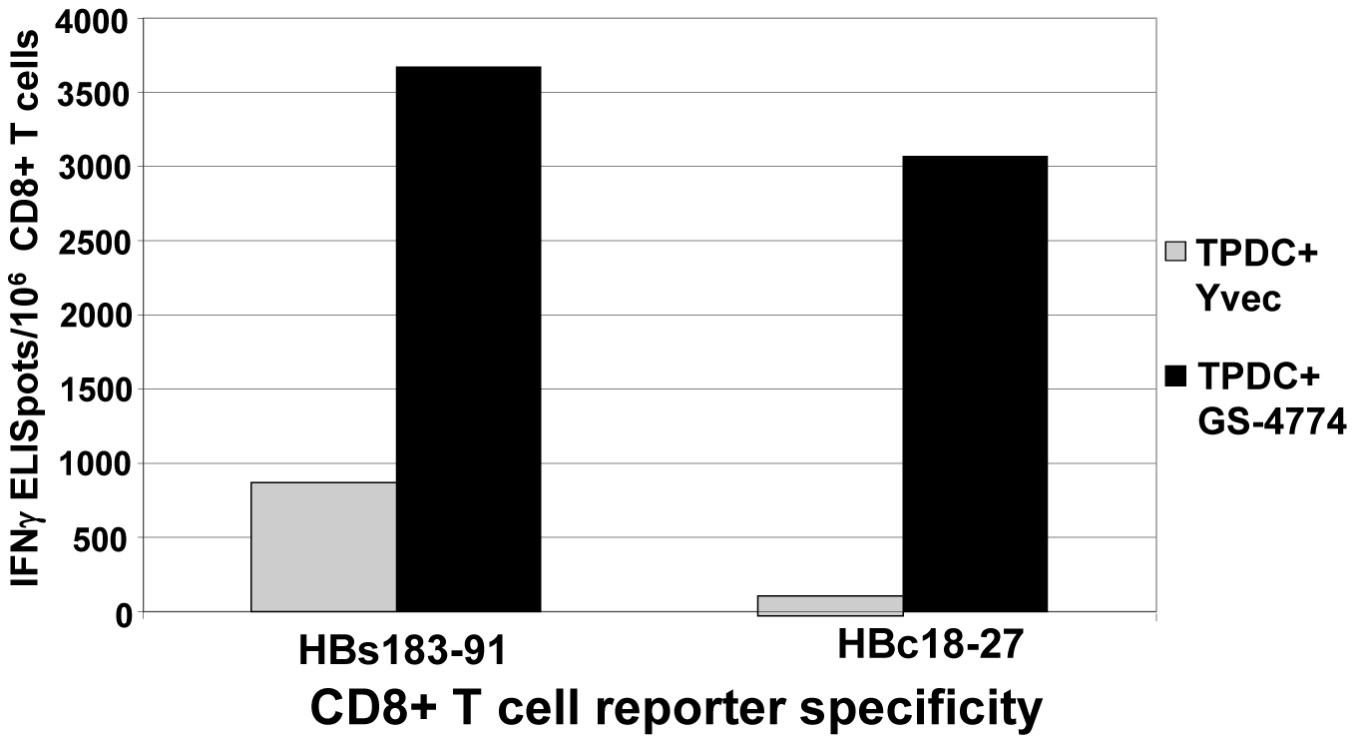
Cross-presentation of HBV antigens to T cells by GS-4774-pulsed human DCs. Tarmogen (GS-4774 or Yvec)-pulsed DCs (TPDCs) were incubated in an IFNγ ELISpot plate with HBs183–91 or HBc18–27 TCR re-directed CD8^+^ T cells at a 2∶1 effector:target ratio (10,000 T cells:5000 moDC). **P- values**, GS-4774 vs. Yvec: HBc18–27, 0.048; HBs183–91: 0.08. The experiment was conducted twice and similar results were obtained each time.

Having shown that GS-4774 triggered DC maturation, we evaluated the capacity of HBV Tarmogens to be processed and their HBV antigens presented to T cells, resulting in activation of HBV-specific CD8^+^ T cells. We evaluated epitopes believed to be important in acute resolved infection: HBs183–91 and HBc18–27 [21] using cells from HLA-A*02∶01-expressing donors. PBMCs from a healthy HLA-A*02∶01 donor were transduced with a plasmid encoding a TCR specific for each epitope and then co-cultured with Tarmogen-pulsed-DCs (TPDCs) in an IFNγ ELISpot assay. GS-4774/Yvec response ratios of 4.2 and 23 for the S (p = 0.08) and Core (p = 0.03) specific T cells, respectively were observed (Fig. 6). The results demonstrated that both epitopes are cross-presented by GS-4774-pulsed DCs, resulting in activation of cognate T cells. Positive control assays using DCs pulsed with the cognate peptides showed antigen-specific T cell stimulation as well (not shown).

### HBV Tarmogen-pulsed DCs from ENGERIX-vaccinated and CHB patients elicit antigen-specific CD4^+^ and CD8^+^ T cell activation

**Figure 7:**
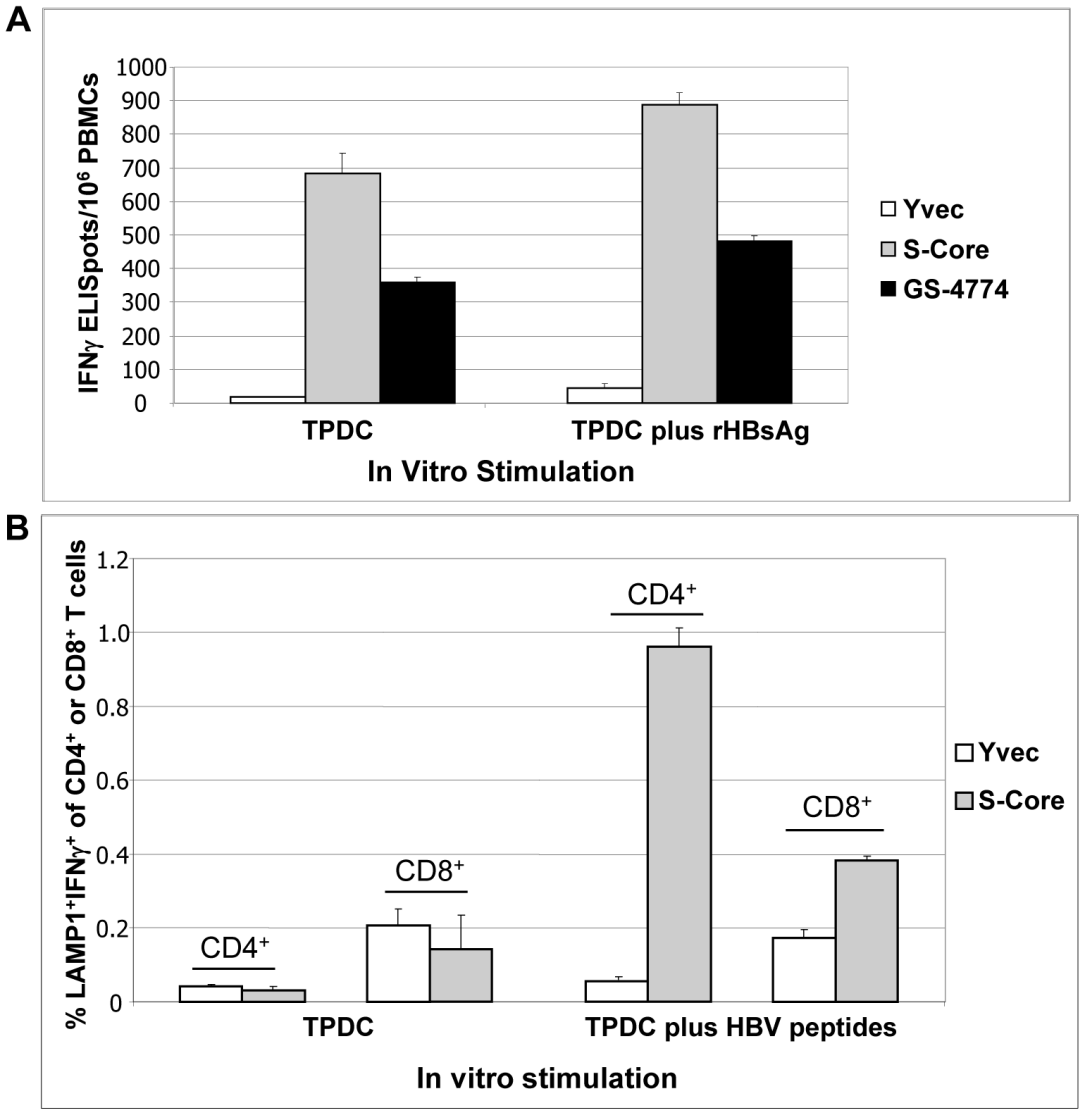
T cell responses to Tarmogen-pulsed DCs in ENGERIX-B vaccinated donor samples. TPDCs were used to stimulate autologous PBMCs over 3 rounds to expand HBV-specific T cells. (A) IFNγ ELISpot response of a donor immunized with Engerix-B six months prior to TPDC expansion. S-Core Tarmogen: identical to GS-4774 except for the absence of HBxAg. **P values** (TPDC plus rHBsAg only): GS-4774 vs. Yvec, 0.0001; S-Core vs. Yvec, 0.0001. (B) ICS staining of donor cells collected 20 years post Engerix-B immunization to evaluate the LAMP1 phenotype of IFNγ-secreting CD4^+^ and CD8^+^ T cells. HBV peptides for CD4^+^ T cell response: PICPGYRWMCLRRFIIFL, FFLLTRILTIPQSLD, SGFLGPLLVLQAGFFLLTR, TRILTIPQSLDSWWTSLNF 10 µg/mL each. HBV peptides for CD8^+^ T cell responses: VLQAGFFLL, FLLTRILTI, LLDYQGMLPV, WLSLLVPFV, SIVSPFIPLL 10 µg/mL each. **P values** (TPDC plus HBV peptides), S-Core vs. Yvec: CD8, 0.0001; CD4, 0.0002.

The finding that epitopes associated with acute HBV protection were cross-presented to T cells by GS-4774-treated DCs prompted us to extend the T cell analysis to subjects with prior HBV Ag exposure and to do so with cells harboring natural rather than heterologous TCRs. For initial experiments, donors previously immunized with the prophylactic alum-based vaccine ENGERIX-B were used, on the basis that they would be expected to possess resting memory HBsAg-specific CD4^+^ T cells and, to a lesser extent CD8^+^ T cells [28], [29]. We hypothesized that these resting memory T cells could be activated and expanded by exposure to the Tarmogen-pulsed DCs (TDPC). PBMCs from these donors (n = 2) were stimulated with DCs that were pulsed with GS-4774 (X-S-Core), the closely related Tarmogen S-Core, or empty vector control yeast (Yvec). After stimulation, T cells were assessed for the production of IFNγ by ELISpot and for one donor, by ICS as well to evaluate lysosomal-associated membrane protein 1 (LAMP1) positivity in IFNγ^+^ CD4^+^ and CD8^+^ T cells. LAMP1 is a degranulation marker that is uniquely expressed on cytolytic T cells [30]. Prior experience in similar assays indicated that optimal responses sometimes required recombinant antigen stimulation concomitant with cytokine accumulation. In a first experiment, ELISpot analysis of stimulated T cells collected from a subject who had completed a full course of Engerix-B injections 6 months prior to sample collection, revealed the presence of activated HBsAg-specific T cells that were producing high quantities of IFNγ. Tarmogen/Yvec response ratios up to 36.6-fold were observed in this experiment, attesting to the high efficiency with which this method expands Ag-specific T cells (Fig. 7A).

A key goal of Tarmogen immunization is to generate HBV-specific cytotoxic T lymphocytes (CTLs) able to kill infected hepatocytes. To determine if the above DC-stimulated T cells possess a cytolytic phenotype, T cell populations were evaluated for expression of the degranulation marker, LAMP1. TPDCs from a different donor, who was immunized in 1992 with ENGERIX-B were incubated with MHC class I or class II specific HBV peptide pools, stained with dye-coupled antibodies recognizing CD4, CD8, LAMP1, and IFNγ, and evaluated by flow cytometry. The results showed that a subset of the HBsAg-specific T cells generated possessed a cytolytic phenotype as demonstrated by LAMP1 expression. The percentage of CD4^+^ T cells that was IFNγ^+^ and LAMP1 positive was similar to that obtained by PBMC incubation with DCs treated with a potent DC activator (CD40L, not shown) and 2.5-fold higher than the corresponding subset of CD8^+^ T cells. The Tarmogen/Yvec response ratio for HBV peptide-pulsed CD4^+^ T cells was 16.8 as compared to 2.2 for the same comparison among CD8^+^ T cells (Fig. 7B and discussion).

**Figure 8:**
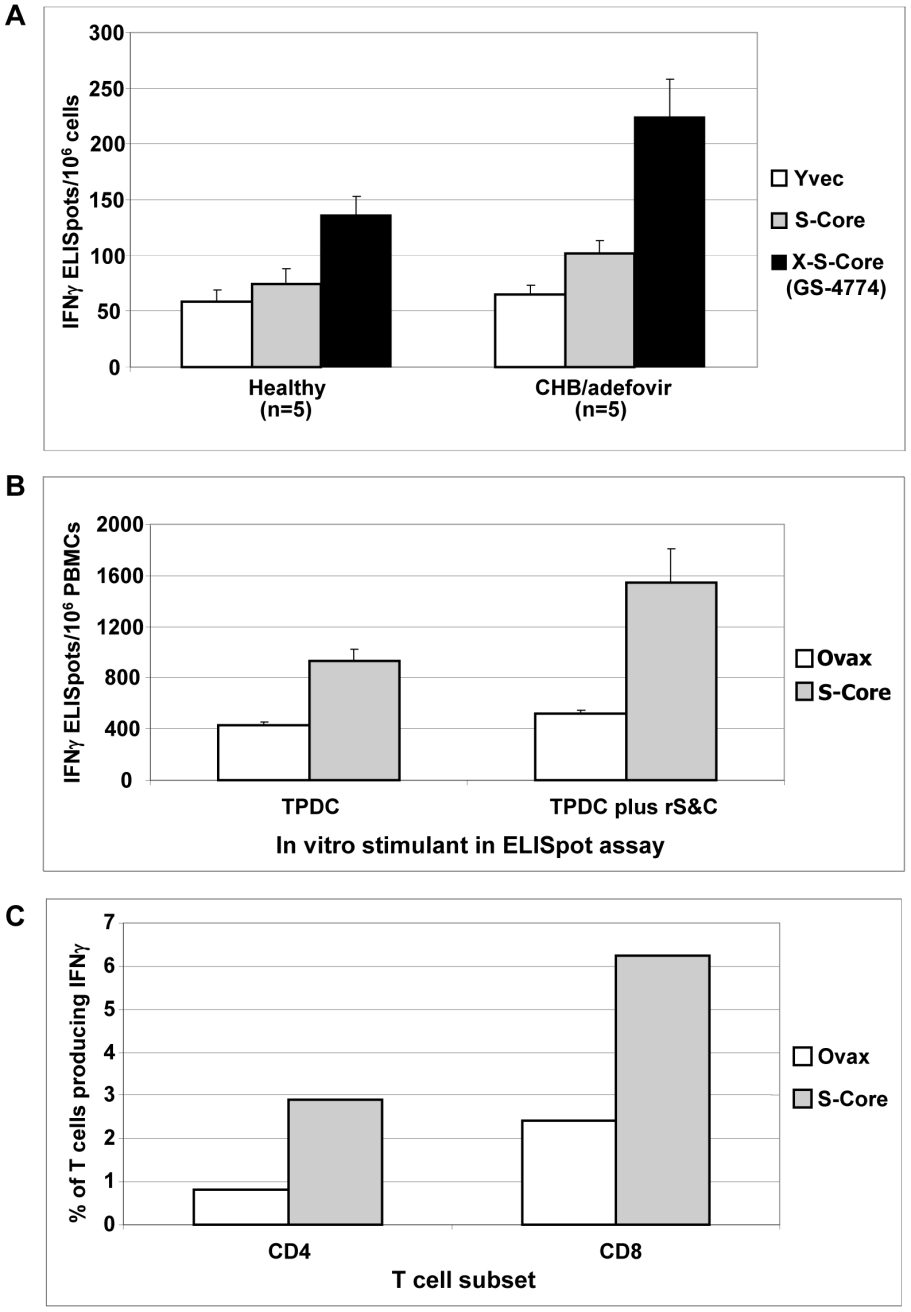
GS-4774-emergent IFNγ responses in healthy subjects or CHB patients on adefovir therapy. (A) TPDCs were used to stimulate autologous PBMCs (two rounds), followed by IFNγ ELISpot assay in the presence of HBV peptide pools. n = 5 subjects/group. **P values** (healthy vs. CHB): GS-4774, <0.0001; S-Core, 0.44; Yvec, 0.95 (B) IFNγ ELISpot response of TPDC-expanded T cells in a chronic HBV donor. **P values**, S-Core vs. Ovax: TPDC, 0.00051; TPDC plus rS&C, 0.0051. (C) Both CD4^+^ and CD8^+^ T cells from the CHB donor produce IFNγ in response to TPDC stimulation. **P values**, S-Core vs. Ovax: CD4, 0.0001; CD8, 0.0001. X-axis label abbreviations: TPDC, expansion with TPDCs only; TPDC plus rHBsAg or HBV peptides, expansion with TPDC followed by 24 hour incubation with rHBsAg or HBV peptides during the ELISpot or ICS assay. X-S-Core, fusion protein expressed in GS-4774.

The prior results were conducted with uninfected healthy individuals who are not anticipated to possess tolerance to HBV antigens nor exhaustion of HBV-specific T cells. To test Tarmogen performance with more clinically relevant samples that are predicted to possess these characteristics, the TPDC assay was conducted with PBMCs from CHB patients taking suppressive antiviral therapy compared to healthy controls. DCs from 5 normal/healthy subjects and 5 CHB donors on active adefovir treatment were prepared and pulsed with Yvec, S-Core, or X-S-Core Tarmogen. The TPDCs were then used to stimulate autologous PBMCs. IFNγ responses were measured during a final stimulation with HBV peptide pools that span the HBV proteome. Both X-S-Core and S-Core pulsed DCs elicited more IFNγ-producing T cells from CHB patients than did DCs pulsed with the Yvec control, and X-S-Core pulsed DCs elicited a significantly greater number of IFNγ-producing T cells from adefovir-treated CHB patients than healthy patients (p<0.0001, Fig. 8A). The GS-4774 response was HBV antigen-specific (GS-4774/Yvec response ratio was 3.4).

To determine if both CD4^+^ and CD8^+^ T cells were induced by TPDCs in CHB cells, another TPDC expansion experiment was done using an additional CHB patient, featuring TPDC expansion followed by in vitro stimulation with mixture of recombinant HBV antigens (HBsAg & HBcAg). An ELISpot assay was first conducted which confirmed that an IFNγ response was mounted by S-Core Tarmogen-pulsed DCs in this subject (3-fold response ratio S-core/Yvec, Fig. 8B). ICS was then carried out on the same population of expanded T cells, using Ab probes specific for CD4, CD8 and IFNγ. Both CD4 and CD8 T cells produced IFNγ and the percentage of cells producing the cytokine was greater for the CD8 subset (6.2% for CD8 cells vs. ∼2.9 for CD4). The activation of both T cell subsets is consistent with the mechanism of action of Tarmogens which includes antigen presentation with MHC class I and class II (Fig. 8C) [14].

## Discussion

The host immune response to HBV is a central determinant of the clinical outcome of infection. Individuals who are able to control or clear HBV infection mount a vigorous, polyclonal antigen-specific, adaptive immune response, whereas those who develop chronic infection display a blunted and inadequate adaptive immune response [31], [32]. The development of a therapeutic vaccine to induce an immune response in CHB patients that parallels that observed in acute self-resolved infection is likely to be a key requirement of successful immunotherapy for this disease. This study provides evidence for the potential of GS-4774 to be used as a HBV- therapeutic vaccine based on 3 lines of evidence: 1) the induction of HBV Ag-specific CD4^+^ and CD8^+^ T cells and anti-tumor responses in multiple mouse models; 2) the activation of T cells that recognize epitopes of known importance in clearance of acute infection, and; 3) the ability to elicit HBV Ag-specific T cell responses *ex vivo* in both healthy and chronically-infected human donor cells.

The immunogenicity of GS-4774 in mice was assessed with *in vivo* functional assays (tumor protection) and with *ex vivo* T cell activation experiments. Using the latter assays which included lymphocyte proliferation, ELISpot, and ICS, we were able to show the induction of CD4 and CD8- based T cell responses that were specific to all 3 antigens of the X-S-Core fusion. Responses to S and Core antigens occurred in 3 different strains of mice including HLA-A2 tg mice wherein HBs14–22 and HBc11–27 specific T cells were activated. A review of HBV epitopes reports that T cells of these specificities are found in 20% and 100% of acute resolved HB patients, respectively, indicating the potential clinical relevance of our HLA-A2 tg mouse data [21]. Responses to HBxAg were not evaluated in this tg strain, but three experiments assessing the immunogenicity of this antigen in GS-4774-immunized mice produced positive results (LPA, Fig. 2; IL-2 ELISpot, Fig. 3D; tumor challenge, Fig. 4). In fact, comprehensive screening of a HBxAg peptide library identified MHC class I and II epitopes in HBxAg that to our knowledge have not been previously identified (Fig. 3D). These new peptide sequences could be useful in future studies of the immune response to HBxAg in mice and their identification provides further justification for inclusion of HBxAg in hepatitis B vaccines.

Whereas the *ex vivo* murine data showed robust and roughly comparable immune responses to all 3 antigens of the X-S-Core fusion, *in vivo* tumor challenge studies showed a notable difference for HBcAg. While the inhibition of all HBV Ag-expressing EL4 targets by immunization with Tarmogen was statistically significant (Table 2), the extent of protection against EL4/Core cells was not as pronounced as for the other targets (Fig. 4B). This could reflect lower immunogenicity of the Core portion of X-S-Core, differential antigen expression in the target, or some combination of these factors. Loss of heterologous antigen expression *in vivo* has been observed by our team and others with various tumor assays (possibly due to chromosome instability or promoter silencing), and this was confirmed to be the case for the EL4/S-Core target in one study by day 11 post-challenge (see results and Fig. S5).

The immunogenicity of the HBV Tarmogens was extended beyond murine models, by using two different human DC assays in which Tarmogen-pulsed DCs were used to activate and/or expand HBV Ag-specific T cells from autologous donors with varying histories of HBV Ag exposure. Using a novel cross-presentation assay, we showed that human T cells expressing TCRs specific for two different epitopes associated with HBV clearance were activated by incubation with GS-4774-treated DCs. This effect was statistically significant for the HBc18-27 specific T cells, which showed a 23-fold higher IFNγ response to GS-4774 than to control yeast (Yvec) after a single incubation with TPDCs. Responses by HBs183–91 specific T cells suggested an antigen-specific response to GS-4774-treated DCs in this population as well, although not significant (p = 0.08). These data indicate that the dominant S and Core epitopes are presented by MHC class I molecules following phagocytosis of GS-4774 by DCs. While cross-presentation of the yeast-expressed antigens in HBV-naive, healthy donor cells confirms that a central component of the vaccine's mechanism is active, the ability of GS-4774 to activate relevant T cells in more clinically meaningful samples was addressed with DC/T cell expansion studies.

These DC co-culture assays featured *ex vivo* stimulation of human PBMCs with autologous Tarmogen-pulsed DCs, and utilized 2 subjects with prior ENGERIX-B immunization plus 6 CHB patients and 5 healthy donors. The stimulation process induced HBV-specific T cells that possessed a cytolytic phenotype on the basis of LAMP1 staining, consistent with our murine tumor protection data suggesting that GS-4774 elicits *in vivo* cytolytic activity (Fig. 7B). While both CD4^+^ and CD8^+^ T cells were found to be LAMP1 positive in this *ex vivo* assay, the phenotype was unexpectedly more pronounced for the CD4^+^ subset, with a Tarmogen/Yvec response ratio of 16.8 (ie, 16.8-fold more IFNγ^+^/LAMP1+/CD4^+^ T cells emerged from S-Core treatment than from Yvec treatment) in samples harvested > two decades after ENGERIX-B exposure. The basis of this apparent CD4+ T cell bias is unknown. Cytolytic CD4^+^ T cells have been reported by others as early as the mid-1990s [33] (and citations therein) and play an important role in the control of some chronic viral infections. For example, cytolytic CMV-specific CD4^+^ T cells are key mediators of CMV infection, and HIV-infected donors possess CD4^+^ perforin^+^ T cells at all stages of the disease [34]. Cytolytic CD4^+^ T cells are also active in chronic HCV and HBV and in HBV/HDV co-infected subjects [35].

Importantly, the results with CHB donors taking antiviral therapy (Fig 8 A–C) demonstrated the ability of HBV Tarmogens to elicit activated, HBV Ag-specific CD4^+^ and CD8^+^ T cells in significant quantities upon stimulation with Tarmogen-pulsed DCs. These findings are noteworthy in light of the prevailing view that CHB is characterized by T cell tolerance and/or exhaustion, and portend the ability of an HBV Tarmogen to restore HBV-specific T cell responses in CHB patients [36]. The response to GS-4774 may have been augmented by adefovir treatment in these patients. Nucleos(t)ide HBV DNA polymerase inhibitors such as adefovir, are associated with an increase in HBV-specific CD8^+^ T-cells and reduction in exhaustion markers (such as PD-1) on CD8+ T-cells, an environment likely to be favorable to Tarmogen activity [11]. Future CHB donor studies to evaluate exhaustion makers in parallel with Tarmogen administration, or experiments in transgenic mice expressing constitutively high levels of HBV antigens could be used to further assess the ability of GS-4774 to elicit cytolytic HBV-specific responses in the context of tolerance to viral antigens and/or T cell exhaustion.

Beyond the ability of GS-4774 to induce cellular immunity to HBV antigens, GS-4774 has other desirable characteristics which are platform-wide and that make it a strong therapeutic vaccine candidate for CHB patients. First, the yeast-based vector does not require extensive purification steps to isolate the antigen or to remove potentially toxic contaminants. Second, unlike many viral vectors, pre-clinical Tarmogen studies in mice and rabbits did not show the emergence of neutralizing antibodies directed against the yeast vector upon repeated administration. In fact, T cell responses to the heterologous yeast-expressed antigen continue to mount over repeated immunizations[15], [16]. Such repeated administration may be of paramount importance where continued long term immunological pressure is required. Others have tested T cell vaccines for the treatment of CHB in animal models, including a recent study of a DNA vaccine prime/adenoviral vector boost method targeting HBcAg in woodchucks and a study in which ISCOMATRIX adjuvant was used to deliver HBV Ag, eliciting potent T cell responses in a Tg mouse model [37], [38]. In addition, the strength and type of immune responses generated by a carcinoembryonic antigen (CEA)-expressing Tarmogen has been compared side-by-side against a viral vaccine system featuring recombinant vaccinia virus priming followed by a fowlpox virus boost, co-delivered with genes encoding co-stimulatory molecule CD80 (B7-1), and adhesion molecules ICAM-1 (CD54) and LFA-3 (CD58), plus the human CEA transgene (abbreviated rV/F-CEA/TRICOM). The two platforms elicited T-cell populations with both shared and unique phenotypic and functional characteristics. For example, T-cell lines generated from yeast-CEA immunization of mice versus lines generated from rV/F-CEA/TRICOM immunization possessed similar levels of CTL activity directed against CEA peptide-pulsed targets, but the yeast-CEA-derived lines elicited notably stronger killing than the viral system when the targets were tumors expressing full length CEA [45]. Although murine comparisons of immunogenicity are unable to predict therapeutic efficacy in the clinic, it is clear that viral vector immunization approaches are constrained to a limited number of injections due to immunological neutralization. Third, many therapeutic vaccine strategies have focused on vaccination with one or more short peptide antigens or have focused on small antigen domains containing primarily or exclusively dominant epitopes. The limited diversity of disease-targeted T cells and frequently an exclusive focus on CD8 T cell induction arising from such approaches is avoided with Tarmogens, by the inclusion of large antigen domains including both dominant and sub-dominant epitopes and by the fact that the processed peptides are presented concomitantly with class I and class II MHC. Lastly, Tarmogens elicit a reduction in the number and activity of Treg cells [17] through a cytokine-mediated shift in the balance of Treg and Th17 cells [39]. This is an inherent property of the yeast vector and one that is anticipated to apply to any Tarmogen including GS-4774. The precise impact of this phenomenon in the setting of cHBV infection is unknown, but an improvement in the clearance of HBV-infected hepatocytes would obviously be a favorable outcome and one that is consistent with recent studies correlating HBV persistence to circulating Treg levels [40], [41].

In this study we describe a new lead candidate therapeutic vaccine that could provide a tangible benefit for CHB patients. Clinical trials featuring GS-4774 immunization of CHB patients whose viral levels are well-controlled with oral nucleos(t)ide HBV polymerase inhibitors are underway and will provide key insights into the potential of the vaccine to improve the rate of durable HBsAg seroconversion with a finite treatment duration regimen.

## Materials and Methods

### Ethics statement

Animal experiments were conducted under the approval of GlobeImmune's Institutional Animal Care and Use Committee (protocol approval number 2011_001C) whose policies are aligned with “The Guide for the Care and Use of Laboratory Animals” (8^th^ edition, published by the National Research Council of the National Academies). All efforts were made to minimize pain and discomfort, and animal numbers were minimized as feasible while retaining sufficient cohort sizes to permit robust statistical analyses. Samples from human subjects with chronic hepatitis B treated with adefovir dipivoxil under National Institute of Allergy and Infectious Diseases (NIAID) clinical trial (NCT00023153) were used for *in vitro* experiments [42]. For these latter samples all subjects signed informed consents approved by the NIAID Institutional Review Board (see also reference [42]). For healthy donor samples used in cross-presentation assays, participants signed written informed consent forms approved by the National University Hospital of Singapore Institutional Review Board, prior to sample collection; see also reference [24].

### Reagents and tissue culture medium

^3^H thymidine was from MP Biomedical. Tissue culture medium (cRPMI-10) consisting of RPMI-glutamax 1640 supplemented with 10% heat-inactivated fetal bovine serum (FBS) (HyClone), 2 mM l-glutamine, 50 µM β-mercaptoethanol, 100 U/ml penicillin, 100 µg/ml streptomycin sulfate (pen-strep) and 10 mM HEPES was used for general cell culture and for all immune assays employing mouse cells.

For cross-presentation assays, RPMI 1640 supplemented with 10% heat-inactivated FBS, 20 mM HEPES, 0.5 mM sodium pyruvate, 100 U/ml penicillin, 100 µg/ml streptomycin, MeM essential and MeM non-essential amino acids, Glutamax, and 55 µM β-ME was used (cRPMI-Hu; see below for cytokine supplementation). Washes were performed with Hank's Balanced Salt Solution (HBSS).

Multi-round DC assays used Aim V base medium containing 1X pen-strep plus 10% human AB serum (cAIMV; InVitrogen). ELISpot kits and cytokines for all experiments were from R&D Systems.

### Tarmogen engineering, preparation, and Ag quantification (see also Methods S1)

HBV genes were cloned behind the *CUP1* promoter in a yeast 2 µm expression vector, and plasmids were transfected into *S. cerevisiae* W303 MATα cells using a lithium acetate/polyethylene glycol approach (Zymo Research). Antigen expression was induced with copper sulfate and yeast cells were heat killed and processed for injection as described previously [16]. Antigen content of GS-4774 lysates was determined by Western blot using interpolation against a standard curve comprised of purified commercial his-tagged recombinant protein.

### Mice and Immunization

Female mice were obtained from Jackson laboratories at 5–7 weeks of age and studies were initiated between 6–10 weeks of age (weight range ∼15–19 g). The strains used were C57BL/6J, BALB/cBy, and HLA-A2 (B6.Cg-Tg(HLA-A/H2-D)2 Enge/J), and *Scid* (B6.CB17-Prkdc*^scid^*/SzJ). The HLA-A2 strain contains the HLA-A*0201 transgene plus wild type (wt) murine MHC alleles. The *scid* mice, which do not have functional T and B cells, were housed and handled under conditions recommended by Jackson Laboratories for all immunodeficient strains. Treatment cohorts ranged in size from 5 to 14 mice/group. Within each experiment, the mice were age- matched and treatments were administered one complete cohort at a time but the order of vaccine treatments delivered was changed on different time points (e.g., weeks) of vaccination. Effect assessment followed a similar regimen. The number of cohorts typically varied between studies but ranged from 2 to 4 and included naive, empty vaccine vector, or test vaccine (Tarmogen)-immunized mice as appropriate. Mice were housed according to Jackson laboratories recommendations for each strain. The Animal Research Facility (ARF) is equipped with an air filtration/ventilation system (Allentown Caging model EP5000E). All cages possessed filtered tops (Allentown caging). Mice were housed at a density maximum of 5 per cage (18×28 cm size) or 10 per cage (23×33 size) with Harlan sani-chip bedding. Food was Harlan # 2018 18% protein rodent chow, and water was from the public drinking water supply (Louisville, CO). The ARF lighting was on a 12 h light/12 h dark automatically timed cycle and the temperature was maintained in the range of 18°C to 23°C. Monthly inspections by the IACUC's lead veterinarian were conducted through the entire course of the experiments described herein.

In some experiments, 2.5 Yeast Units (YU: 10^7^ yeast cells) of Tarmogen was injected subcutaneously (s.c.) at each of two sites: flank, and scruff between the shoulder blades (Method A). In others, 1 YU of Tarmogen was injected s.c. at each of four sites: both inner thighs and above each shoulder blade to target the inguinal, axillary, and subclavicular lymph node beds (Method B). The latter method generates higher frequency T cell responses in some assay systems [15]. Immunizations were performed in a biological safety cabinet in GlobeImmune's ARF between 0700 and 1100 in most experiments; timing of injections was held consistent from week to week.

### Preparation of splenocytes and lymph node cells for *in vitro* Ag stimulation

Mice were euthanized by CO_2_ asphyxiation and splenocytes and lymph node (LN) cells were prepared as previously described [27]. ACK lysis was not performed for LN cell preparations. Unless otherwise indicated in Figure legends, spleens or LNs from 5 Tarmogen-vaccinated mice were pooled prior to in vitro stimulation. For this type of *ex vivo* work, cohort sizes were shown in the present study and in prior published studies [16] to result in reproducible immune response data.

### In vitro stimulation for ELISpot, LPA, and intracellular cytokine staining

Two ×10^5^ splenocytes or LN cells were incubated in a 200 µL volume of cRPMI medium plus or minus added HBV Ag in round bottom 96-well tissue culture plates. Incubation with antigens occurred at 37°C in a humidified 5% CO_2_ chamber for 4 to 7 days depending on the experiment (see Results for incubation times associated with each experiment).

### CD4^+^ T cell enrichment

CD4^+^ T cells were prepared from splenocytes by negative Magnetic Activated Cell Sorting (MACS) per Miltenyi Biotech instructions (CD4 T cell isolation kit II).

### Lymphocyte proliferation assay (LPA)

After 4 days of in vitro stimulation, 1 µCi/well of ^3^H-thymidine was added in 20 µL fresh media to each well for an additional 20 hours (h) at 37°C in a humidified CO_2_ incubator. Cells were processed for scintillation counting as described previously [16].

### IFNγ and IL-2 ELISpot assay

Splenocytes or LN cells were placed into in vitro stimulation with antigen for 4 days. Cell suspensions were mixed and 200,000 cells/well were transferred to IFNγ/IL-2 dual color or IFNγ single analyte murine ELISpot plates for 24 h (4 to 6 replicate in vitro stimulation wells per treatment). Plates were developed per R&D Systems instructions and spot counting was performed by Cellular Technologies, Ltd. (CTL). Experimental outcomes were determined by comparisons of average spot number differences (averages, with ANOVA evaluation across treatment groups).

### Intracellular Cytokine Staining (ICS; see also Methods S1)

Briefly, lymphocytes were incubated with peptide for 7 days to expand cognate T cells, and then Ficoll-fractionated to remove dead cells. Cultures were incubated with the same peptide sequence plus GolgiStop (BD Biosciences) for 5 h at 37°C. Cells were stained with dye-coupled antibodies recognizing CD4 and CD8, then fixed, permeablized and stained with antibodies recognizing IL2, IFNγ, or TNFα. Flow cytometry was used to evaluate the intracellular accumulation of these cytokines in each T cell subset.

### Endpoint measures for murine ex vivo work

Experimental outcomes for ELISpot, LPA, and ICS assays were determined by average spot number differences, average ^3^H-thymidine incorporation differences, or subset analysis differences for CD4^+^ and CD8^+^ T cell subsets (ANOVA evaluation across treatment groups was conducted where applicable; see Statistics section below for details).

### Creation of EL4 tumor lines expressing HBV X, Core, and S-Core antigens (see also Methods S1)

EL4 cells were obtained from ATCC (no. TIB-39). EL4 tumor lines were transfected or virally transduced with mouse codon-optimized HBV genes matched in sequence to the HBV antigens within GS-4774, with the exception of: i) the presence of a polyubiquitin tag to accelerate processing of the S-Core target, and 2) the HBxAg tumor target contains two tandem copies of the X region expressed in the vaccine. All genes were expressed from the Ubiquitin C promoter. EL4/core and EL4/X were created by lentiviral transformation whereas EL4/S-Core was created by lipofectamine 2000-mediated transfection followed by G418 drug selection. Clonal cell lines were established by standard limiting dilution methods.

### Tumor challenge assays

For adoptive transfer tumor protection, wt C57BL/6 mice were immunized by method A and one week later, splenocytes were harvested and preparations (2×10^7^ cells/mouse) were injected i.p. into naive 5 week old *scid* mice. Twenty four hours later, 300,000 EL4 tumor cells expressing a HBV S-Core fusion protein (EL4-S-Core cells) were implanted s.c. (right ribcage) and tumor growth was measured at Day 7 post-challenge.

For all other tumor challenge assays featuring the S-Core target, C57BL/6 mice were immunized by method A and one week later the mice were s.c. challenged in the ribcage with 300,000 tumor cells. For challenge experiments with HBcAg and HBxAg-expressing targets, immunization was done by method B and the number of tumor cells used for challenge was 30,000 and 90,000, respectively. Tumor diameter was measured daily with digital calipers. For these studies the number of mice ranged from 8 to 14 mice per group (total per experiment: 16 to 42), which was shown in the present study and prior GlobeImmune experience to be required to achieve statistical significance by ANOVA.

### Monocyte-derived DC generation and cross-presentation (see also Methods S1)

CD14^+^ monocytes (MN) were isolated using CD14 microbeads (Miltenyi Biotech) and cultured in cRPMI-Hu medium (composition in supportive information). Day six monocyte-derived DC (moDC) were prepared and cultured alone or activated with 10∶1 Tarmogen:moDC. The moDCs were washed, stained with Abs recognizing DC maturation markers, and analyzed by flow cytometry.

For cross-presentation assays, immature moDC produced from a healthy HLA-A*02∶01^+^ donor was incubated with Tarmogens (10∶1, Tarmogen:moDC) for 24 h. MoDCs were transferred to anti-IFN-γ antibody-coated ELISpot plates, and co-cultured at a 2∶1 effector:target ratio (10,000 T cells:5000 moDC) with TCR-redirected CD8^+^ T cells specific for HBc18–27 (FLPSDFFPSV) or HBs183–91 (FLLTRILTI) for 24 h. IFN-γ ELISpot assays and data acquisition were performed per standard methods (CTL, Inc.).

HBV-specific TCR-redirected CD8^+^ T cells were generated from a healthy HLA-A*02∶01 negative donor as previously described [24].

### *Ex vivo* multi-round stimulation of human donor PBMCs with Tarmogen-pulsed DCs (see also Methods S1)

Donor PBMCs were used to prepare DCs by treating adherent cells in plastic tissue culture flasks with GM-CSF and IL-4 as described previously [26]. DCs were pulsed with Tarmogen for 24-48 h resulting in mature DCs which were detached, irradiated, and co-cultured with autologous PBMCs for 7 days (one round of expansion). A total of 2 rounds (CHB data, Fig 7C) or three rounds (remainder of Fig. 7) were conducted. Following expansion, suspension lymphocytes were subject to IFNγ ELISpot or ICS plus or minus recombinant antigen or peptide pool stimulation.

### CHB patient data and reagents

All CHB patients were on lamivdudine 100 mg daily, previously tested positive for the HBV YMDD mutation daily and were initiated on 10 mg adefovir dipivoxil for 48 weeks [42]. Peripheral blood mononuclear cells from baseline (prior to initiation of adefovir) and at the end of 48 weeks of treatment were used to perform in vitro stimulation. Stimulation of DC-expanded T cells was with HBV peptide pools spanning the entire HBV proteome (2 µg/ml each peptide per million PBMCs).

### Statistics

Statistical significance for *ex vivo* assays was determined by ANOVA (Microsoft XLSTAT 2008). For tumor challenge assays, hazard ratios (hr) were calculated using a Cox proportional hazards model (QST consultations) or log-rank analysis, and associated p-values were calculated by log rank tests from a Kaplan-Meier analysis in Graphpad Prism v6.0.

## Supporting Information

Figure S1 HBxAg peptide sequences used in IL2 ELISpot screen. Seventy-six overlapping HBX peptides were screened in a search for novel class I (9-mer) and class II (15-mer) - restricted epitopes in GS-4774-immunized BALB/c mice.
(TIF)

Figure S2 ELISpot response to all HBxAg peptides tested in GS-4774 immunized BALB/c mice. Splenocytes from 10 GS-4774 or Yvec-immunized mice were pooled and stimulated with 7 µM of 44 different 9-mer peptides and 32 different 15-mer peptides spanning the X Ag portion of the X-S-Core fusion protein expressed in GS-4774. After a 4 day in vitro stimulation, a 24 h IL2 ELISpot assay was conducted. Positive responses: ELISpot counts in GS-4774-immunized mice is >40 spots per million splenocytes, with a GS-4774/Yvec response ratio of >2.5. *, Positive responding peptides. **P values**, GS-4774 vs. Yvec: peptide # 18 VLHKRTLGL, 0.005; peptide # 49 AHQFLPKVLHKRTLG, 0.061; peptide # 58 HKRTLGLSAMSTTDL, 0.034. Error bars: s.e. for quadruplicate stimulations of the pooled immune cells.
(TIF)

Figure S3 Example of flow cytometric data for Th1 cytokine responses in CD8+T cells isolated from GS-4774 (X-S-Core)-immunized C57BL/6 mice. ICS was used to assess the production of IFNγ, IL-2, and TNFα by CD8^+^ T cells in the presence of peptide HBs190-197 (VWLSVIWM). Ovax: control Tarmogen expressing chicken ovalbumin. Gating strategy: Upper left panel, live cell gate; Lower left panel; gating on CD8^+^B220^−^CD4^−^MHC class II^−^ T cells.
(TIF)

Figure S4 S-Core but not Yvec Tarmogen induces protective immunity against challenge with EL4/S-Core but not EL4/Ovalbumin (Ova) tumors. C57BL/6 mice were immunized with S-Core Tarmogen, Yvec, or nothing (naive) by Method A and one week later, splenocytes were harvested and adoptively transferred to naive *scid* mice. 24 h later, the *scid* mice were s.c. challenged with 300,000 EL4-S-Core or EG7.Ova (EL4/Ova) tumor cells. Tumor diameter (mm) was measured 10 days post-challenge. Error bars, s.e. **P values:**see Figure.
(TIF)

Figure S5 EL4 tumors lose S-Core mRNA expression by day 11 post challenge. Tumors that escaped Tarmogen-mediated killing have lost S-Core mRNA expression by day 11 post challenge. Tumors that were not eliminated by Tarmogen vaccination were excised from mice at day 11 post-challenge, snap-frozen in liquid nitrogen, and total RNA was isolated and subjected to real time PCR to evaluate S-Core mRNA quantity relative to samples comprised of known percentages of S-Core-expressing cells (“mixing curve”). Example X-Axis labeling: “Ovax2”, mouse # 2 of Ovax immunization group;”X-S-Core2”, mouse # 2 of X-S-Core immunization group. EL4+EL4/S-Core: In vitro cultured, untransfected EL4 cells (EL4) were mixed with EL4/S-Core-expressing cells at the indicated ratios prior to RNA isolation.
(TIF)

Figure S6 S-Core Tarmogen induces maturation of human monocyte-derived dendritic cells (moDCs). CD14^+^ monocytes were isolated from healthy donors and cultured with GM-CSF + IL-4 for 6 days to generate immature moDCs which were then incubated for 24 h with 10 Tarmogens per 1 moDC. The moDCs were stained with dye-coupled antibodies recognizing CD80, CD83, CD86 HLA-DR, or HLA-A, B, & C and evaluated by flow cytometry.
(TIFF)

Methods S1 Additional methodology for selected procedures. Procedural details for Tarmogen engineering, intracellular cytokine staining, creation of murine tumor cell lines, and dendritic cell manipulations are provided in this methods supplement.
(DOC)
