# Supplementary figures and images for: A Whole Recombinant Yeast-Based Therapeutic Vaccine Elicits HBV X, S and Core Specific T Cells in Mice and Activates Human T Cells Recognizing Epitopes Linked to Viral Clearance
Source: PLoS One. 2014 Jul 22;9(7):e101904. doi: 10.1371/journal.pone.0101904 (PMC4106793; doi:10.1371/journal.pone.0101904)

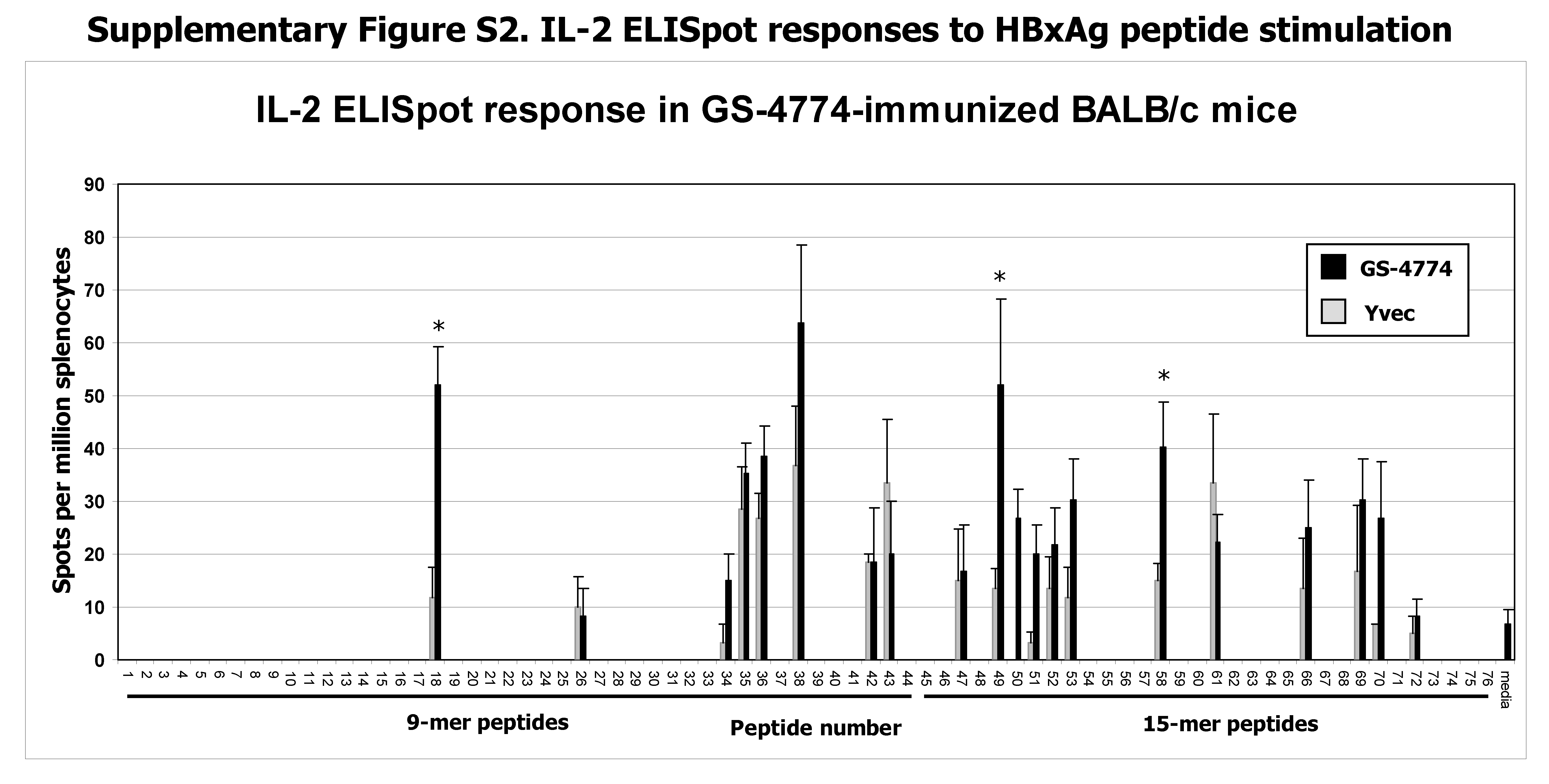

Supplement: Figure S2 — ELISpot response to all HBxAg peptides tested in GS-4774 immunized BALB/c mice. Splenocytes from 10 GS-4774 or Yvec-immunized mice were pooled and stimulated with 7 µM of 44 different 9-mer peptides and 32 different 15-mer peptides spanning the X Ag portion of the X-S-Core fusion protein expressed in GS-4774. After a 4 day in vitro stimulation, a 24 h IL2 ELISpot assay was conducted. Positive responses: ELISpot counts in GS-4774-immunized mice is >40 spots per million splenocytes, with a GS-4774/Yvec response ratio of >2.5. *, Positive responding peptides. P values, GS-4774 vs. Yvec: peptide # 18 VLHKRTLGL, 0.005; peptide # 49 AHQFLPKVLHKRTLG, 0.061; peptide # 58 HKRTLGLSAMSTTDL, 0.034. Error bars: s.e. for quadruplicate stimulations of the pooled immune cells. (TIF) [file pone.0101904.s002.tif]

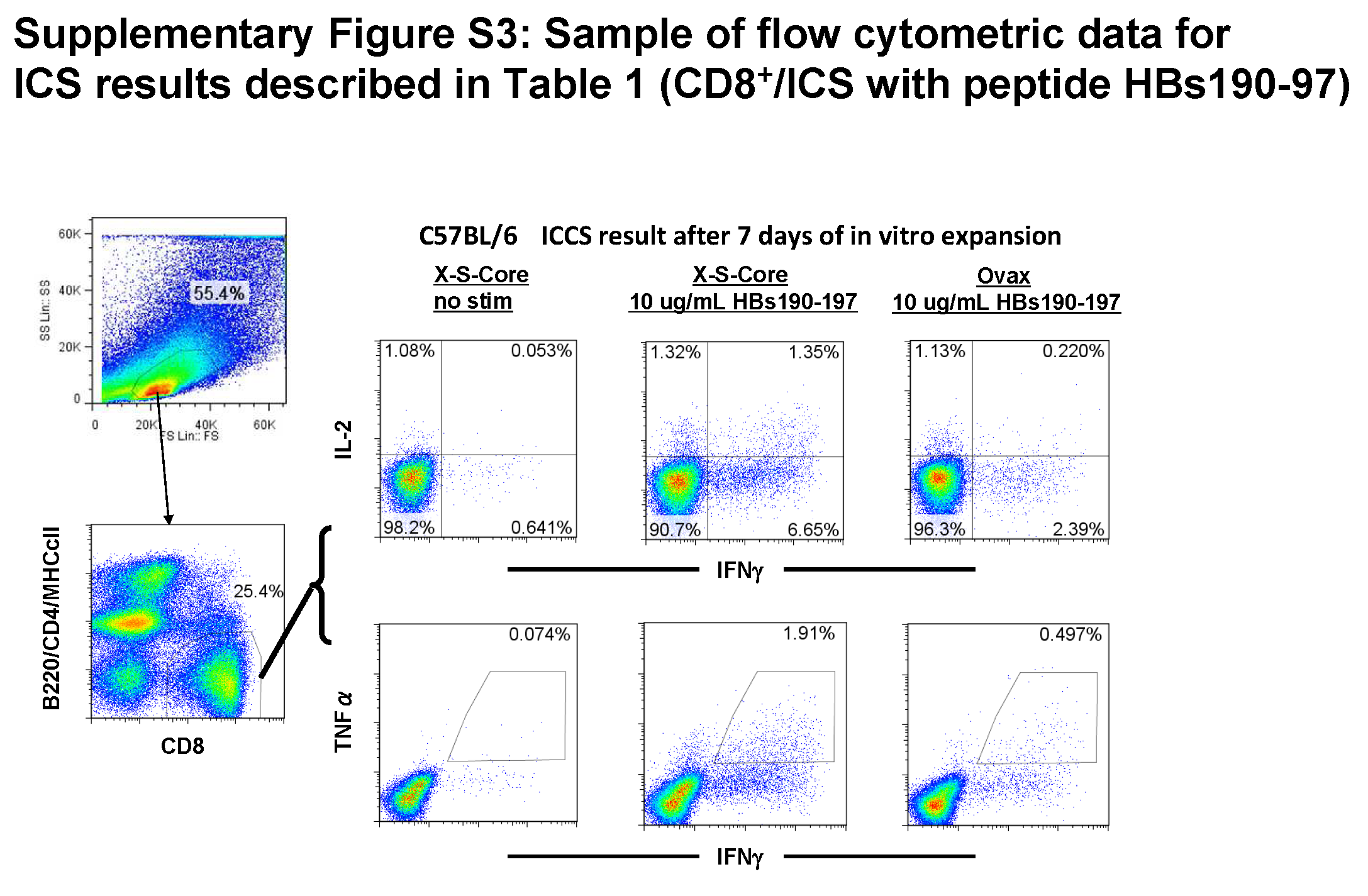

Supplement: Figure S3 — Example of flow cytometric data for Th1 cytokine responses in CD8+T cells isolated from GS-4774 (X-S-Core)-immunized C57BL/6 mice. ICS was used to assess the production of IFNγ, IL-2, and TNFα by CD8+ T cells in the presence of peptide HBs190-197 (VWLSVIWM). Ovax: control Tarmogen expressing chicken ovalbumin. Gating strategy: Upper left panel, live cell gate; Lower left panel; gating on CD8+B220−CD4−MHC class II− T cells. (TIF) [file pone.0101904.s003.tif]

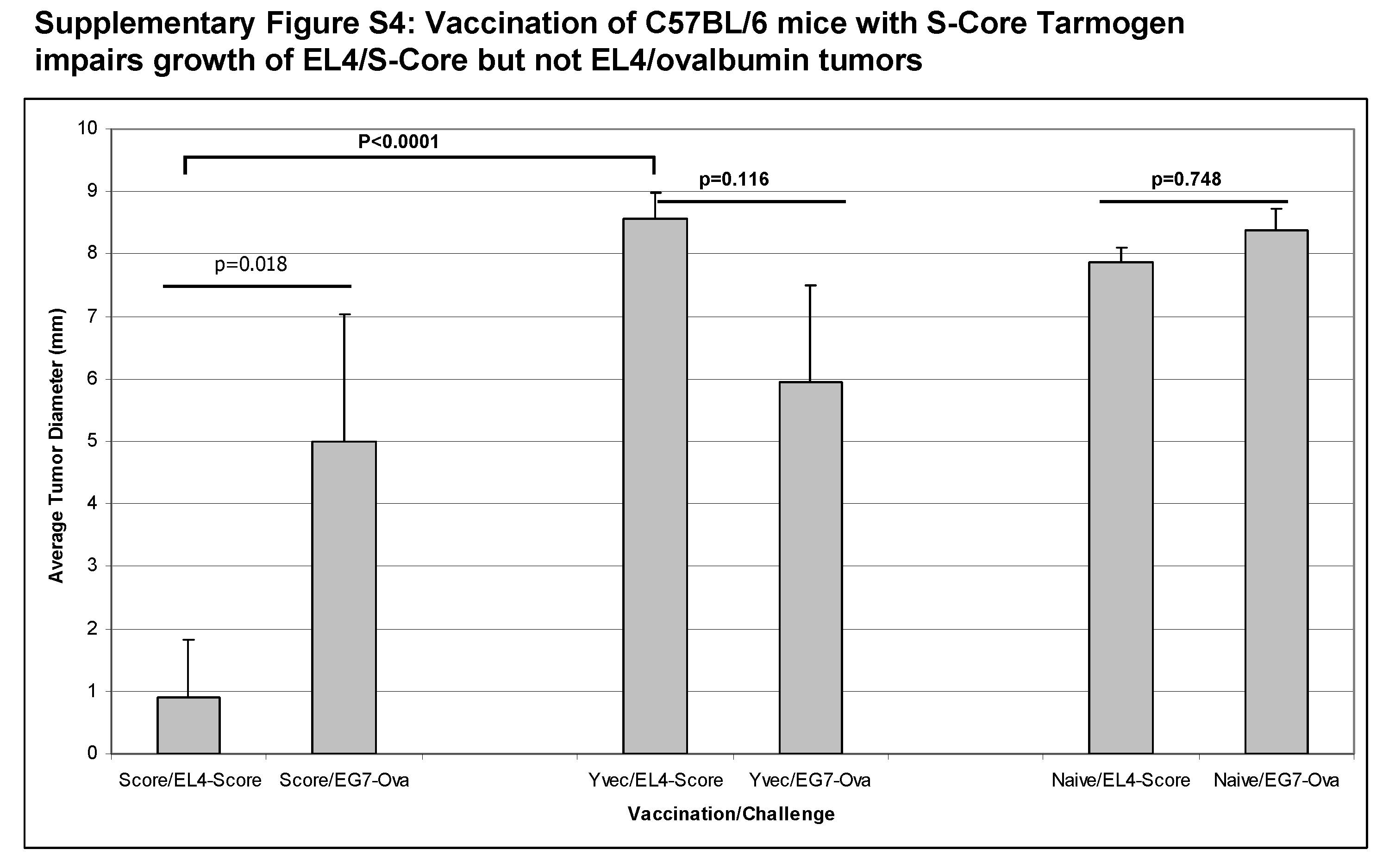

Supplement: Figure S4 — S-Core but not Yvec Tarmogen induces protective immunity against challenge with EL4/S-Core but not EL4/Ovalbumin (Ova) tumors. C57BL/6 mice were immunized with S-Core Tarmogen, Yvec, or nothing (naive) by Method A and one week later, splenocytes were harvested and adoptively transferred to naive scid mice. 24 h later, the scid mice were s.c. challenged with 300,000 EL4-S-Core or EG7.Ova (EL4/Ova) tumor cells. Tumor diameter (mm) was measured 10 days post-challenge. Error bars, s.e. P values:see Figure. (TIF) [file pone.0101904.s004.tif]

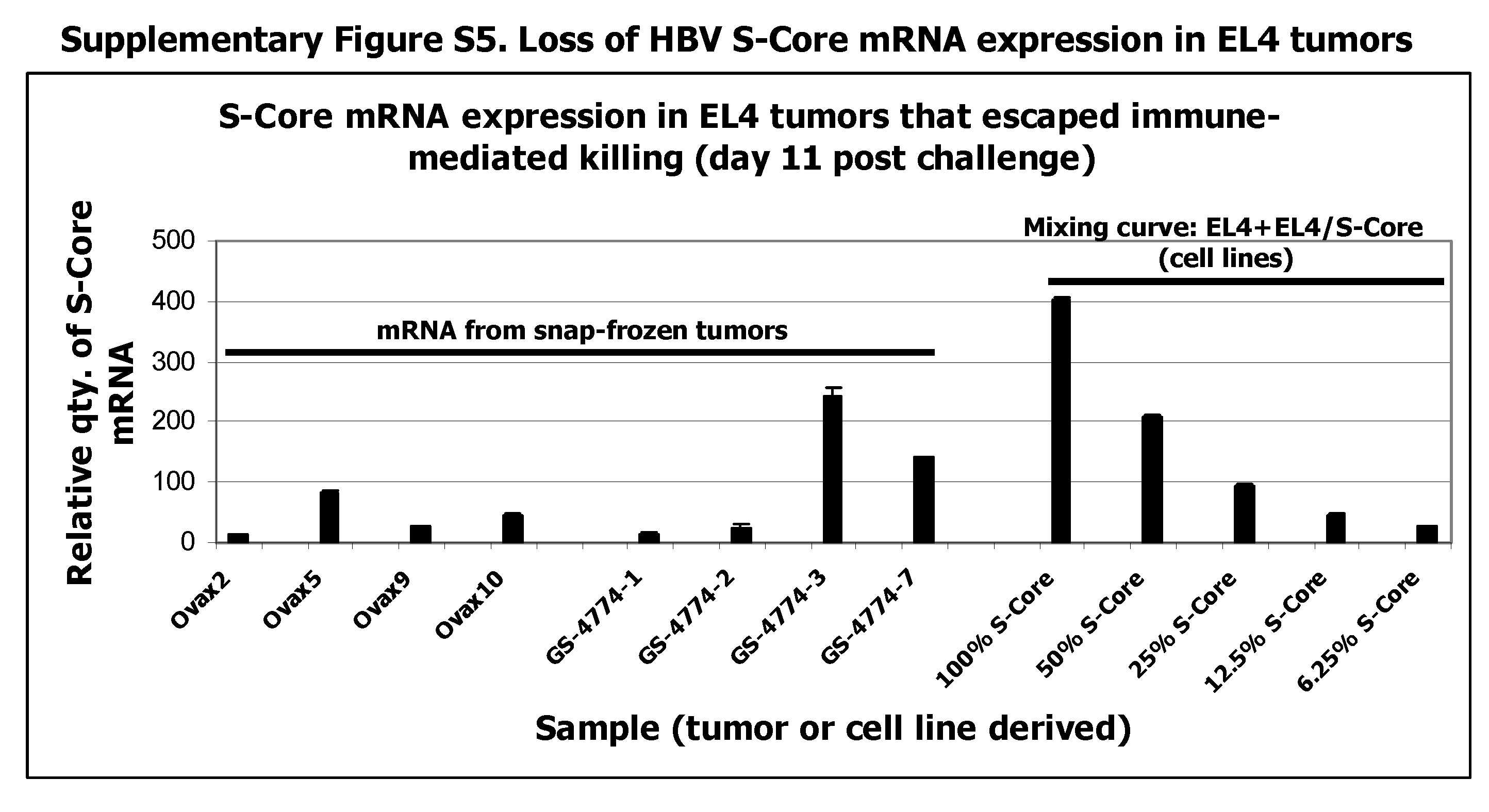

Supplement: Figure S5 — EL4 tumors lose S-Core mRNA expression by day 11 post challenge. Tumors that escaped Tarmogen-mediated killing have lost S-Core mRNA expression by day 11 post challenge. Tumors that were not eliminated by Tarmogen vaccination were excised from mice at day 11 post-challenge, snap-frozen in liquid nitrogen, and total RNA was isolated and subjected to real time PCR to evaluate S-Core mRNA quantity relative to samples comprised of known percentages of S-Core-expressing cells (“mixing curve”). Example X-Axis labeling: “Ovax2”, mouse # 2 of Ovax immunization group;”X-S-Core2”, mouse # 2 of X-S-Core immunization group. EL4+EL4/S-Core: In vitro cultured, untransfected EL4 cells (EL4) were mixed with EL4/S-Core-expressing cells at the indicated ratios prior to RNA isolation. (TIF) [file pone.0101904.s005.tif]

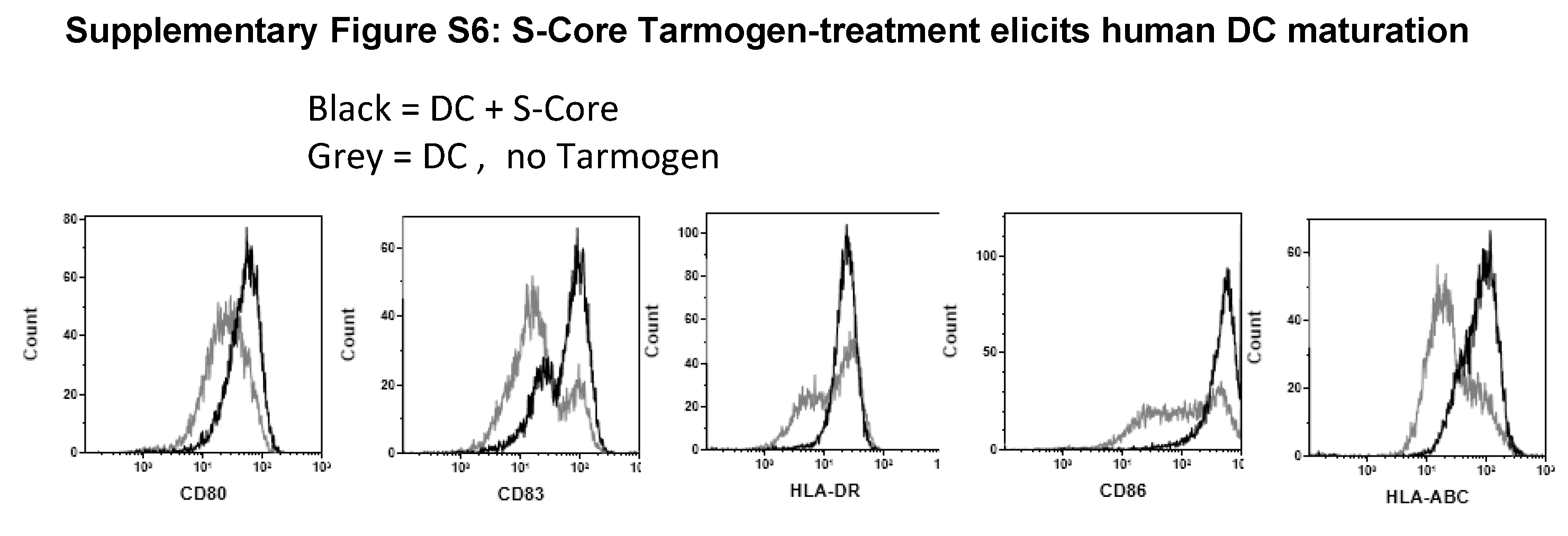

Supplement: Figure S6 — S-Core Tarmogen induces maturation of human monocyte-derived dendritic cells (moDCs). CD14+ monocytes were isolated from healthy donors and cultured with GM-CSF + IL-4 for 6 days to generate immature moDCs which were then incubated for 24 h with 10 Tarmogens per 1 moDC. The moDCs were stained with dye-coupled antibodies recognizing CD80, CD83, CD86 HLA-DR, or HLA-A, B, & C and evaluated by flow cytometry. (TIFF) [file pone.0101904.s006.tif]
