# Supplementary material for: A Whole Recombinant Yeast-Based Therapeutic Vaccine Elicits HBV X, S and Core Specific T Cells in Mice and Activates Human T Cells Recognizing Epitopes Linked to Viral Clearance
Source: PLoS One. 2014 Jul 22;9(7):e101904. doi: 10.1371/journal.pone.0101904 (PMC4106793; doi:10.1371/journal.pone.0101904)
Supplement: Methods S1 — Additional methodology for selected procedures. Procedural details for Tarmogen engineering, intracellular cytokine staining, creation of murine tumor cell lines, and dendritic cell manipulations are provided in this methods supplement. (DOC) [file pone.0101904.s007.doc]

**Methods S1 (Methods supplement)**

Tarmogen engineering, preparation, and antigen quantification

HBV X, S, and Core genes were produced synthetically (DNA2.0) and were cloned as X-S-Core or S-Core fusion configurations behind the copper-inducible *CUP1* promoter in a yeast 2 µm expression vector. Consensus sequences were chosen for each gene based on the computational alignment of 322 individual isolates of the three antigens from HBV genotype D [1]. The amino acid sequence of S-Core is identical to the corresponding region the X-S-Core construct. Yeast cells were growth to a density of 2.5 x 10^7^ cells/mL (2.5 YU; one YU=1 X 10^7^ cells) in synthetic complete medium containing 6.7 g/L yeast nitrogen base, 15g/L glucose, and 0.04 g/L of adenine, tryptophan, and histidine, then induced with 0.375 mM copper sulfate for 3h at 30ºC (250 rpm) to initiate synthesis if the X-S-Core fusion protein. Cells were washed in PBS, heat inactivated at 56ºC for 1h, then washed three times in PBS to remove residual copper. Antigen content was quantified as described previously [2] using Western blot analysis with a his-tag specific monoclonal antibody (Ab) probe.

Intracellular Cytokine Staining (ICS)

Splenocytes were incubated with 1 µg/mL peptide for 7 days, with the addition of 20 U/mL of recombinant murine IL-2 on days 2 and 5. Cells were then ficoll-fractionated to remove dead cells, and incubated with 10 µg/mL peptide and Golgi stop for 5h at 37ºC. Cells were then stained with dye-coupled antibodies recognizing CD8 (APC-Cy7), CD4 (eFluor 450 or PerCP), B220 (PerCP) and MHC class II (PerCP) surface antigens, fixed, permeabilized and stained with antibodies recognizing IL-2 (Alexa Fluor 647), IFNγ (PE) or TNFα (Alexa Fluor 488). Flow cytometry was used to evaluate the intracellular accumulation of these cytokines in CD8+CD4-B220-MHCII- or CD4+CD8-B220-MHCII- T cell populations.

Creation of EL4 tumor lines expressing HBV X, Core, and S-Core Ags

*Murine cell lines:* C57BL/6-derived EL4 tumor cells and EL4 cells stably expressing chicken ovalbumin (E.G7-Ova) were obtained from the American Type Culture Collection (ATCC).

*EL4/X Ag Target (lentiviral system)*

DNA encoding the 60 amino acid fragment of HBxAg that is expressed in the X-S-Core Tarmogen was fused in tandem to create a cassette expressing two copies of this gene which was then placed under control of the ubiquitin C promoter. The open reading frame was codon-optimized for expression in mouse. The plasmid was packaged into the cell line 293FT using a commercial kit (InVitrogen) and antigen expression was determined by Western blot featuring a rabbit polyclonal anti-X Ab (Abcam). Supernatants (2 mL) from the 293FT packaging reaction were used to infect EL4 cells which were then placed in fresh medium for 24h. Transductants were cloned by limiting dilution in cRPMI+6 µg/mL Blasticidin, and clones were expanded in the same medium for ~ 20 days. HBx mRNA and protein expression were tested in clonal isolates by RT-PCR and Western blot.

*EL4/Core Ag Target (lentiviral system)*

The open reading frame encoding HBcAg was codon-optimized for expression in mouse cells and the EL4/Core line was otherwise constructed as described for EL4/X.

*EL4/S-Core fusion Ag*

A ubiquitin tag was fused in frame to the N-terminus of a S-Core fusion that is sequence-matched to the S-Core sequence domain of X-S-Core. The purpose of the ubiqutin tag is to accelerate proteasomal degradation of the target Ag, a system intended to generate more efficient class I MHC Ag presentation [3]. This cassette was placed downstream of the polyubiquitin C promoter in plasmid pup-neo which carries a G418 drug resistance gene, and the gene was transfected into EL4 cells with Lipofectamine 2000. Transfectants recovered for 1 day in the absence of G418 and were then incubated for 1 week in 1 mg/mL G418. Expansion and expression testing of clonal isolates was conducted as for EL4-X except that an anti-his tag mAb was used in Western blotting.

Monocyte-derived DC generation and cross-presentation

CD14^+^ monocytes (MN) were isolated from healthy donors using CD14 microbeads (Miltenyi Biotech). Purified MN were cultured in cRPMI-Hu. Monocyte-derived DC were made by culturing CD14^+^ MN in cRPMI-Hu plus 50 ng/ml (1500 U/ml) IL-4 and 50 ng/ml (750 U/ml) GM-CSF (R&D systems) for 6 days. Half of the medium was removed after 3 days and replaced with cRPMI-Hu containing cytokines. Day six moDC were cultured for 24h in medium alone or activated with 10:1 Tarmogen:moDC. The MoDCs were then washed twice with HBSS and stained with HLA-DR-PE-Cy7, CD80-PE, CD83-PerCp-Cy5.5 (BD Biosciences), CD86-APC and HLA-A,B,C (Biolegend) specific antibodies for 30 min on ice to measure moDC activation/maturation. Cells were acquired on a FACS Canto flow cytometer and data analyzed using Kaluza (Beckman Coulter).

For cross-presentation assays, 200,000 immature moDC produced from healthy HLA-A*02:01^+^ donors were loaded with Tarmogens (10:1, Tarmogen:moDC) for 24 h. MoDCs were washed and transferred to anti-IFN-γ Ab coated ELISpot plates, and co-cultured at 2:1 effector:target ratio (10,000 T cells:5000 moDC) with TCR-redirected CD8^+^ T cells specific for HBc18-27 (FLPSDFFPSV) or HBs183-91 (FLLTRILTI) for 24 h. DC pulsed with 5 μg/ml of HBc18-27 or HBs183-91 peptide served as positive controls for T cell activation. IFN-γ ELISpot was performed as described previously using 5 μg/ml of 1-D1K Ab for capture and 0.5 μg/ml 7B6-1-Biotin plus Streptavidin-ALP for detection (Mabtec). ELISpot plates were developed and analyzed using CTL Immunospot® analyzer.

HBV-specific TCR-redirected CD8^+^ T cells were generated by introducing HBV-specific T cell receptor genes into the lymphocytes of healthy donors as previously described [4]. Following transduction, CD8^+^ T cells were negatively selected using the CD8 T cell isolation kit (Miltenyi Biotech) and cryopreserved until use. Cells were typically 98% CD8^+^ and 30 to 40 % of the CD8^+^ T cells were specific for the HLA-A2-bound HBc18-17 or HBs183-91 peptides.

*Ex vivo* stimulation of human donor PBMCs with Tarmogen-pulsed DCs

*Preparation of yeast-fed DCs*

Ten million viable PBMCs were incubated in one well of a 6-well plate for 2 hours in cAIMV. The non-adherent cells were discarded and the adherent cells gently rinsed once with cAIM-V to remove non-adherent cells. Five mL of cAIMV containing 100 ng/mL recombinant human GM-CSF (R&D systems) plus 20 ng/mL recombinant human IL-4 were added to the adherent cells and the plate was incubated for 5 days at 37ºC.

The number of DCs was estimated by trypan blue dye exclusion and Tarmogen was added to DCs at a ratio of 1 yeast cell to 1 DC and the plate was returned to the incubator for an additional 40h. The adherent, yeast-fed cells were rinsed twice with calcium- and magnesium-free PBS and then incubated for 15 minutes with non-enzymatic cell dissociation buffer at 37ºC (InVitrogen). The Tarmogen-pulsed DC (TPDCs) were detached from the plastic by gentle pipeting and then irradiated (30 Gy) and stored on ice.

*Stimulation of PBMCs with yeast-fed DCs (3 rounds), and cytokine assays*

Ten to 20 million frozen autologous PBMCs were thawed, washed in pre-warmed cAIMV, counted, and combined with irradiated yeast-pulsed DCs at a ratio of 1:10 (DC:PBMC) in a T-75 tissue culture flask. The mixture was incubated for 3 days at 37ºC and then recombinant human IL-2 was added to a final concentration of 20 U/mL for 4 additional days. This process comprised 1 round of stimulation (1 week). The suspension cells from the round 1 stimulation were transferred to a new flask and the stimulation process was repeated with freshly pulsed DCs (round 2). The stimulation process was repeated once more, using the suspension cells from round 2. On day 6 of the 3rd round, fresh autologous PBMCs were thawed, washed in cAIMV, and incubated with 3 µg/mL of recombinant HBV Ag for 24h to allow antigen uptake and presentation by antigen presenting cells (APCs). The pulsed APCs were washed 1X in cAIMV, counted, and added to the 3rd round-DC-stimulated effector population for 36h in an IFNγ ELISpot plate (200,000 cells/well; 6 wells/condition). ELISpots were counted by Cellular Technology, Ltd.

For ICS, an aliquot of cells collected after round 3 was treated with Golgi Stop (BD Cytofix-Cytoperm kit) in the presence of HBV peptides for 5h at 37ºC. Cells were then stained with anti-human CD8/APC-Cy7 Ab and anti-human CD4/PerCP-Cy5.5 Ab. After fixation with 4% paraformaldehyde, cells were permeablized and stained with a PE-coupled anti-human IFNγ Ab as well as a PE-Cy7-coupled Ab recognizing human IL-4. Cells were analyzed by flow cytometry to determine the percentages of T cell subsets producing IL-4 and IFNγ.  In some experiments, an Alexa Fluor 488-coupled Ab recognizing CD107a (LAMP1) was added concomitant with CD4 and CD8 marker staining. All Ab dilutions followed manufacturer's recommendations.

Real time PCR to detect S-Core mRNA in excised tumors

A 30 µL sized portion of each tumor was excised and snap-frozen in liquid nitrogen (LN2). Cells were grown to a fine powder under LN2 with a mortar and pestle and the powder was transferred to 1 mL of TRI reagent (Ambion). Total RNA was immediately isolated using Ambion's Ribopure total RNA kit with elution into 40 uL; all samples were quantified by A_260_ and adjusted to equal concentration. cDNA was then prepared from the normalized RNA template by random priming using a high capacity cDNA synthesis kit (Applied Biosystems) per the manufacturer's protocol. Three µL of 1:10 diluted cDNA was subjected to real time PCR with 1X complete SYBR green master mix plus 1 µL each of forward and reverse S-Core-specific PCR primers (5 µM each) in a 20 µL volume. The PCR thermal profile was as follows. A 20 second incubation at 95ºC followed by 40 cycles of the following: 95ºC for 15 seconds then 60ºC for 1 min (Applied Biosystems 7500 Fast real time PCR system). Results were analyzed with 7500 Fast SDS software v1.4.

**References for Methods Supplement**:

1. Yu H, Yuan Q, Ge S-X, Wang H-Y, Zhang Y-L, et al. (2010) Molecular and phylogenetic analyses suggest an additional hepatitis B virus genotype “I”. PLoS One 5: e9297.

2. Haller A, Lauer GM, King TH, Kemmler C, Fiolkoski V, et al. (2007) Whole recombinant yeast-based immunotherapy induces potent T cell responses targeting HCV NS3 and Core proteins. Vaccine 25: 1452–1463.

3. Andersson H, Barry M (2004) Maximizing antigen targeting to the proteasome for gene-based vaccines. Mol Ther 10: 432–446.

4. Gehring AJ, Xue S-A, Ho ZZ, Teoh D, Ruedl C, et al. (2011) Engineering virus-specific T cells that target HBV infected hepatocytes and hepatocellular carcinoma cell lines. J Hepatol 55: 103–110.
